# Targeted DNA methylation in human cells using engineered dCas9-methyltransferases

**DOI:** 10.1038/s41598-017-06757-0

**Published:** 2017-07-27

**Authors:** Tina Xiong, Glenna E. Meister, Rachael E. Workman, Nathaniel C. Kato, Michael J. Spellberg, Fulya Turker, Winston Timp, Marc Ostermeier, Carl D. Novina

**Affiliations:** 1 Department of Chemical and Biomolecular Engineering, Johns Hopkins University, Baltimore, MD 21218 USA; 2 Department of Cancer Immunology and Virology, Dana-Farber Cancer Institute, Boston, MA 02215 USA; 3 Department of Medicine, Harvard Medical School, Boston, MA 02115 USA; 4 Broad Institute of Harvard and MIT, Cambridge, MA 02141 USA; 5 Department of Biomedical Engineering, Johns Hopkins University, Baltimore, MD 21218 USA; 6 Boston University, Boston, MA 02215 USA

## Abstract

Mammalian genomes exhibit complex patterns of gene expression regulated, in part, by DNA methylation. The advent of engineered DNA methyltransferases (MTases) to target DNA methylation to specific sites in the genome will accelerate many areas of biological research. However, targeted MTases require clear design rules to direct site-specific DNA methylation and minimize the unintended effects of off-target DNA methylation. Here we report a targeted MTase composed of an artificially split CpG MTase (sMTase) with one fragment fused to a catalytically-inactive Cas9 (dCas9) that directs the functional assembly of sMTase fragments at the targeted CpG site. We precisely map RNA-programmed DNA methylation to targeted CpG sites as a function of distance and orientation from the protospacer adjacent motif (PAM). Expression of the dCas9-sMTase in mammalian cells led to predictable and efficient (up to ~70%) DNA methylation at targeted sites. Multiplexing sgRNAs enabled targeting methylation to multiple sites in a single promoter and to multiple sites in multiple promoters. This programmable de novo MTase tool might be used for studying mechanisms of initiation, spreading and inheritance of DNA methylation, and for therapeutic gene silencing.

## Introduction

DNA methylation is important in many biological processes including genome imprinting, X-chromosome inactivation, chromosome stability, gene regulation^1–4^ and differentiation and development^1, 5–9^. High levels of cytosine methylation at CpG sequences in promoter and enhancer regions are often associated with transcriptional silencing by poorly-understood mechanisms. Many reports demonstrate an inverse correlation between gene expression and the amount of promoter methylation^10^. Several reports demonstrate that methylation of single CpG sites are associated with decreased gene expression^11–17^. It is still unclear if promoters simply require a certain density of methylation for gene silencing, if there are specific sites necessary for gene silencing, if particular sites can initiate spreading of methylation, or if methylation alone can nucleate the formation of higher-order chromatin structuring, which enables effective gene silencing. The study of the mechanisms of initiation, spreading and inheritance of DNA methylation are hindered without tools to efficiently and reproducibly direct methylation patterns to precise sites in model systems.

A common strategy for targeting DNA methylation to genomic sites involves end-to-end fusion of a DNA binding motif and a cytosine DNA methyltransferase (MTase), typically human DNMT3a or bacterial enzymes^18, 19^. The first attempts developing this strategy used zinc fingers (ZFs) as the DNA binding motif^19^. With the advent of the CRISPR-Cas9 systems^20–22^, five groups have recently reported targeted DNA methylation using the catalytic domain of DNMT3a (or DNMT3a-DNMT3L) fused to catalytically-deactivated Cas9 (dCas9) in mammalian cells^23–27^. A key feature of dCas9 as a DNA-binding motif is its ability to bind specific sites determined by single-strand guide RNA (sgRNA) sequences that match the protospacer and protospacer adjacent motif (PAM) on the DNA; a feature that obviates the need to design different ZFs or TALEs for each CpG site targeted. Studies using dCas9-DNMT3a fusions demonstrate that sgRNAs can direct DNA methylation to a target promoter, can be multiplexed, and can down-regulate target genes^23–27^. However, dCas9-DNMT3a fusions were not designed to precisely target single CpG sites and presumably recruit endogenous proteins, including additional DNMT3a/DNMT3L MTase complexes to the region^27–29^ which would serve to amplify DNA methylation near dCas9 binding sites and confound the ability to understand the effects of individual methylation events. Additionally, DNMT3a proteins also have large regulatory domains which have been shown to interact with histone modifying enzymes such as histone deacetylase^30^ and histone methyltransferases^31^. New, highly-specific programmable DNA MTases must be developed to determine the precise relationship between DNA methylation and changes in gene expression. Therefore, we sought to develop a new system in which methylation could be limited to small, predictable regions in the genome.

Here we report the development of a dCas9-derived bacterial split MTase (sMTase) system for targeting DNA methylation to specific CpG sites in mammalian genomes. We use the DNA adjacent to the target site as a template for locally assembling an active form of an otherwise ineffective split methyltransferase to link dCas9 binding and methylation activity^32–34^. We build upon our previous work in which an M.SssI CpG methyltransferase was split between residues 272 and 273 and each fragment was fused to a ZF designed to bind sites flanking the target CpG site^33^. We designed fusion proteins of *Streptococcus pyogenes* dCas9 and the M.SssI fragments and developed design rules for efficient methylation in both *E. coli* and human cells. This programmable sMTase system enables precise and multiplex control over CpG methylation in human cells.

## Results

### Design of the targeted sMTase

We chose to leave the N-terminal fragment of M.SssI (MN) untethered to a DNA-binding domain, in contrast to our ZF-sMTases. We hypothesized that this design would still facilitate targeting of DNA methylation to sites in the genome. As with our ZF-sMTases, when the dCas9 domain is not bound to DNA, the two M.SssI fragments would lack sufficient stability or affinity for each other to efficiently methylate DNA. When the dCas9 is bound to the sgRNA-determined DNA site, localization of the MC domain would increase its stability via interactions with the CpG site or induce its folding in cooperation with MN. Additionally, localizing only one of the M.SssI fragments negates the need to find two flanking PAM sites near a CpG site, thus increasing the number of CpG sites that are targetable.

**Figure 1:**
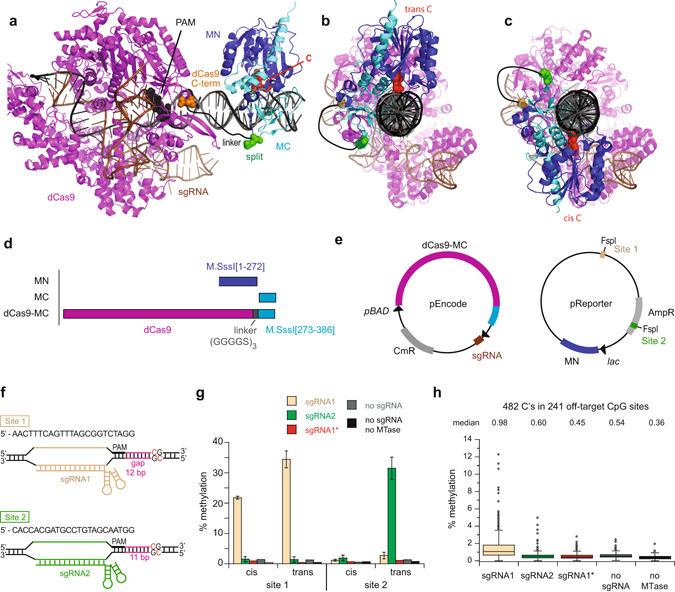
RNA-guided CpG methylation in *E. coli*. (**a**) Model of dCas9 and M.SssI bound to the same DNA at a gap of 11 base pairs between the PAM and the target CpG site. Model was built by superimposing the crystal structures of DNA-bound dCas9 (4UN3)^37^ and the M.SssI homolog M.HhaI (2HR1)^55^ on the same molecule of B-DNA. M.HhaI is bound to the trans strand relative to the PAM site. Red indicates the methylated cytosine, which is flipped out of the dsDNA. A hand-drawn black line represents the 15-amino acid linker between the C-terminus of dCas9 (orange) and the N-terminus of MC (green). The linker was not explicitly modeled. (**b**,**c**) Side views of the model with M.HhaI bound to the trans or cis strand at a gap length of 11 base pairs. (**d**) Protein constructs used in *E. coli* studies. (**e**) Two-plasmid system for expressing dCas9-MC/MN and sgRNA and assaying methylation in *E. coli*. pReporter contains two target sites for methylation in which the CpG site is imbedded within an FspI site. (**f**) DNA sequence near the two target CpG sites for methylation indicating the PAM site (bold) and its distance from the CpG site (the gap). Full sequence of sgRNAs can be found in Supplementary Table S1. (**g**) Frequency of methylation at site 1 and site 2 on pReporter caused by dCas9-MC/MN as assayed by high-throughput bisulfite sequencing. The sgRNA expressed is indicated as is the no methylation control. sgRNA1* indicates that sgRNA1 was expressed, but that the site 1 protospacer and PAM sequences were scrambled. Values are the mean and the error bars are the standard deviation of three independent cultures. (**h**) Frequency of methylation at all off-target cytosines in CpG sites in pReporter as assayed by high-throughput bisulfite sequencing. The pattern of methylation on the plasmids can be found in Supplementary Figure S2.

We constructed models of dCas9 and a bacterial MTase bound to a CpG site on the same DNA molecule to guide the design of our targeted sMTase (Fig. 1a). We adopted the nomenclature “cis” for the PAM/protospacer-containing DNA strand and “trans” for the sgRNA-complementary strand to define the two cytosines in a CpG site relative to the dCas9 binding site. We varied the distance between the PAM and the CpG site (the ‘gap’) and modeled the MTase bound to the cis and the trans strand of the CpG site (Fig. 1b,c). These models suggested that fusion of the C-terminus of dCas9 to N-terminus of the M.SssI[273–386] fragment (dCas9-MC) was the best geometric arrangement to target a CpG site near a PAM sequence **(**Fig. 1d **)**. We estimated that a 15 amino acid flexible linker [(GGGGS)_3_] between the two domains would suffice for connecting the C-terminus of dCas9 and the N-terminus of MC bound at target sites up to about 20 base pairs of DNA away from the PAM site. Our models indicated that a gap of about 11 base pairs between the PAM sequence and the target CpG site would be optimal for initial testing, as the C-terminus of dCas9 and the N-terminus of MC fusion sites would be on the same side of the DNA helix regardless of which strand the MTase was bound to (Fig. 1b,c). Thus, the linker between dCas9 and MC would not be required to wrap around the DNA to methylate either C in the CpG site, potentially enabling efficient methylation of both the strands.

### RNA-programmed DNA methylation in *E. coli*

Compared to eukaryotic cells, *E. coli* have many advantages for in-depth characterization of targeted DNA methylation systems due to their ease of use, inexpensive reagents, shorter doubling times, and lack of endogenous CpG methylation. We constructed a two-plasmid system for assaying targeted methylation activity in *E. coli* (Fig. 1e). Plasmid pReporter encoded MN and two prescribed CpG target sites for testing methylation (site 1 and site 2) with NGG PAM sequences 12 or 11 bps away, respectively. Both sites were contained within a methylation-sensitive restriction enzyme site, FspI, presenting a convenient test for methylation. We created two sgRNAs to target these sites and expressed them individually on plasmid pEncode along with the dCas9-MC fragment (Fig. 1e,f). We engineered the sgRNA1 to be a single-strand version^20^ of a previously-described 20 nucleotide guide sequence^35^, whereas sgRNA2 was designed to target methylation to a known FspI site in the β-lactamase gene (site 2) which had a PAM site a suitable distance away. As controls, we also constructed a version of pEncode lacking any sgRNA and a version of pReporter lacking methylation site 1.

To test the methylation activity of dCas9-MC/MN, we cotransformed the pEncode and pReporter plasmids into *E. coli* and induced expression of both MTase fragments. We assessed methylation on pReporter by restriction enzyme protection assays at the two FspI sites (Supplementary Fig. S1) and high-throughput bisulfite sequencing of the entire plasmid (Supplementary Fig. S2). These experiments demonstrated significant sgRNA-targeted methylation (Fig. 1g,h). We observed that targeted DNA methylation required expression of dCas9-MC/MN, the presence of sgRNA, and the presence of the sgRNA’s protospacer on the DNA. The level of methylation at a target cytosine was as high as 34.4 ± 2.8% (Fig. 1g), and the median percent methylation at cytosines in the 241 off-target CpG sites was never higher than 1.0% (Fig. 1h). dCas9-MC/MN guided by sgRNA1 had a 58-fold preference for methylating target site 1 over the median off-target site. Changing the guide RNA from sgRNA1 to sgRNA2 redirected targeted methylation from site 1 to site 2, causing a 130-fold preference for site 2 over the median off-target site. We confirmed that splitting the methyltransferase facilitates targeting, as dCas9 fused to full-length M.SssI methylated site 1 and site 2 equally at about 100% efficiency when sgRNA1 was expressed (Supplementary Fig. S3). These initial experiments demonstrated that our dCas9-sMTase system showed high specificity to target sites in *E. coli*, and that use of a split-methyltransferase was essential to limit off target effects.

### Rules for sgRNA design for targeted methylation

**Figure 2:**
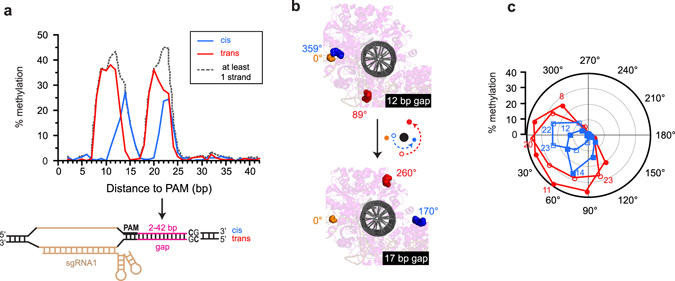
The frequency of methylation at the target CpG site varies periodically with its distance from the PAM site. (**a**) Frequency of methylation for cis and trans strands. The dotted line indicates the fraction of the target sites that are methylated on at least one strand assuming that strand-specific methylation is independent. The cis strand is defined as the strand with the PAM and protospacer. (**b**) Side views of the model at good (12 base pair) and poor (17 base pair) gap lengths for methylation. Orange, C-terminus of dCas9; blue, N-terminus of MC bound to the cis strand; red, N-terminus of MC bound to the trans strand. dCas9 is shown in the background. The MTase is not shown except for its N-terminus of MC. Angles relative to the position of the C-terminus dCas9 are indicated. (**c**) Polar graph indicating the fraction methylated on the cis (blue) and trans (red) strands as a function of the angle as defined in (**b**). The colored numbers indicated the length of select gap lengths and open symbols indicate data points in the second rotation around the DNA helix. The graph shows how efficient methylation only happens with the N-terminus of MC is positioned on the same side of the DNA as the C-terminus of dCas9.

At the target site, sgRNA1 directed methylation to both cis and trans strands, albeit at different levels, but sgRNA2 effectively caused methylation only on the trans strand (Fig. 1g). As site 1 and site 2 had different gap lengths between the PAM and the CpG site, this difference suggested that the spatial relationship between the PAM site and each strand of the CpG site determined methylation efficiency. This observation led us to systematically explore how gap length affected methylation efficiency on both strands. We determined dCas9-MC/MN’s ability to methylate each C in a CpG site positioned 2 to 42 base pairs from the PAM site (Fig. 2). We found that methylation did not occur if the CpG site was less than 8 base pairs or more than 25 base pairs away from the PAM. The level of DNA methylation between these distances oscillated with a period of ~11 base pairs, which is one turn of DNA. Peak targeting, as calculated by the likelihood of having at least one strand methylated, occurred at 12 base pairs and 22–23 base pairs away from the PAM (Fig. 2a). In general, dCas9-MC/MN methylated the trans strand better than the cis strand, suggesting a geometric constraint on cis strand methylation.

The periodicity and strand-dependent differences in methylation efficiency can be explained by our structural model. Methylation of the both strands requires that MC binds to the CpG site in two different orientations relative to dCas9 and these orientations rotate around the DNA as the gap length increases (Fig. 2b). Methylation is favored when the fusion sites of MC to dCas9 are located on the same side of the double helix (Fig. 2c), presumably because the linker does not have to wrap around the DNA. At certain gap lengths, the fusion sites of dCas9 and MC sit on opposite sides of the DNA helix preventing methylation, but as the gap lengths continue to increase, the fusion sites will once again be on the same side of the DNA allowing for methylation (Fig. 2b). The gap distance where this occurs differs depending on if the sMTase is bound to the cis or trans strand resulting in the off-set peak methylation sites for the two strands **(**Fig. 2a **)**.

### Mechanism of off-target methylation

An unaddressed question in studies of dCas9-DNMT3a in mammalian cells is how off-target sites become methylated and what governs which off-target sites are methylated. The answers to such questions are important for assessing the limitations of targeted DNA methyltransferase tools and interpreting the results of targeted methylation experiments. In theory, off-target DNA methylation for dCas9-MC/MN can occur by four non-exclusive mechanisms: (1) Once the MC and MN domains assemble at a target site, the sMTase acts processively on the DNA to methylate other sites downstream. (2) The sMTase fragments assemble to methylate DNA without dCas9 being bound to DNA. (3) The dCas9 domain binds to DNA at off-target sites to methylate nearby CpGs (i.e. dCas9 specificity is the issue). (4) Off-target DNA methylation occurs with dCas9 bound at the intended site, and methylation at off-target sites is proportional to a site’s proximity to the target site, which would be dependent on plasmid 3D topology in the cell.

**Figure 3:**
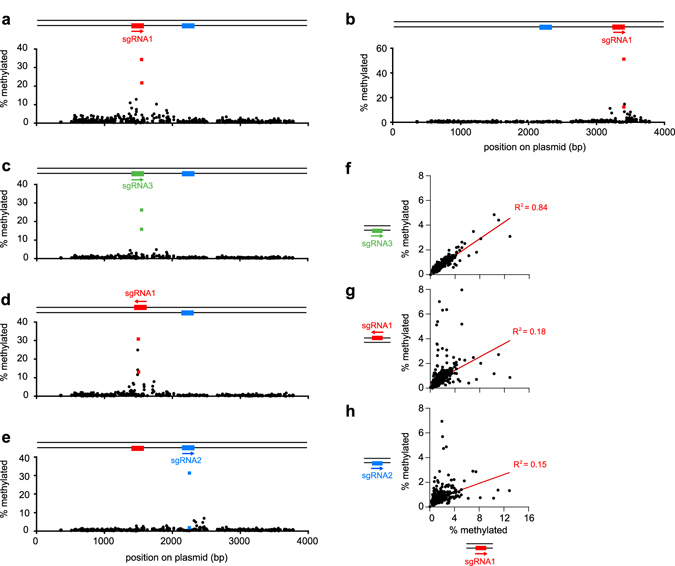
The location and level of off-target DNA methylation depends upon the location of the targeted sgRNA protospacer in the plasmid and its relative orientation. (**a–e**) Methylation of CpG sites as a function of position on the plasmid and sgRNA (arrows) as determined by high throughput bisulfite sequencing. The frequency of methylation at the target sites CpG (colored squares) and the 482 off-target sites (black circles) is shown. The presence and location of an sgRNA’s binding site on the plasmid is indicated by a colored rectangle on the schematic of dsDNA above the graph the x-axis. Enlarged log-scale graphs of the fraction methylated plotted with error bars (along with additional negative controls) are provided in Supplementary Fig. S2. (**f**) Correlation of off-target methylation frequencies when site 1 is replaced with site 3 on the plasmid. (**g**) Non-correlation of the off-target methylation levels when comparing inverted protospacer sites for the same sgRNA. (**h**) Non-correlation of off-target methylation frequencies when the dCas9-MC/MN is directed to different sites by different sgRNAs.

To test these theories, we quantified DNA methylation at the target site and the ~240 off-target CpG sites in pReporter for different combinations of sgRNAs and sgRNA protospacers. Our results indicate that off-target DNA methylation primarily occurs when the dCas9 domain is bound at its target site. Although off-target sites with the highest level of DNA methylation are located within ~400 bp of the sgRNA’s protospacer, dCas9-MC/MN does not function processively because off-target DNA methylation is low at many sites near the target CpG site (Fig. 3 and Supplementary Fig. S2). Functional assembly of MN/MC without dCas9 bound to DNA cannot be the major mechanism because moving site 1 to a different location on the plasmid changed the pattern of off-target methylation (Fig. 3a,b) and methylation levels at off-target CpG sites using sgRNA1 and sgRNA2 showed little correlation (*R* ^2^ = 0.15) (Fig. 3h). Additionally, removing sgRNA1’s protospacer from the plasmid decreased the mean level of sgRNA1-guided DNA methylation at off-target sites by 7-fold (Supplementary Fig. S2) and there was little correlation between DNA methylation levels at off-target sites between the two experiments (*R* ^2^ = 0.16–0.23) (Supplementary Fig. S4). Binding of dCas9 at unintended sites cannot be the major mechanism because replacing site 1 with site 3 (targeted by sgRNA3) left the pattern of off-target DNA methylation relatively unchanged (*R* ^2^ = 0.84) (Fig. 3f), but inverting site 1 changed the pattern of off-target DNA methylation (Fig. 3g).

In summary, the location and degree of off-target DNA methylation depends upon the location of the sgRNA protospacer in the plasmid and its relative orientation (and thus the relative orientation of the DNA-bound dCas9). Significant levels of off-target DNA methylation require both an sgRNA and its corresponding protospacer, but the same pattern of non-target methylation will occur with a different sgRNA/protospacer combination as long as the protospacer is at the same site on the plasmid. The simplest explanation for these observations is that off-target sites are methylated primarily by dCas9-MC/MN with its dCas9 domain bound at its intended sgRNA-target site. We conclude that the pattern of off-target methylation levels reflects the topology of the plasmid DNA in the cell and the degree to which CpG sites elsewhere on the same plasmid molecule find themselves proximal to the dCas9-MC bound at its target site, perhaps due to plasmid supercoiling.

### RNA-programmed targeting of methylation in human cells

**Figure 4:**
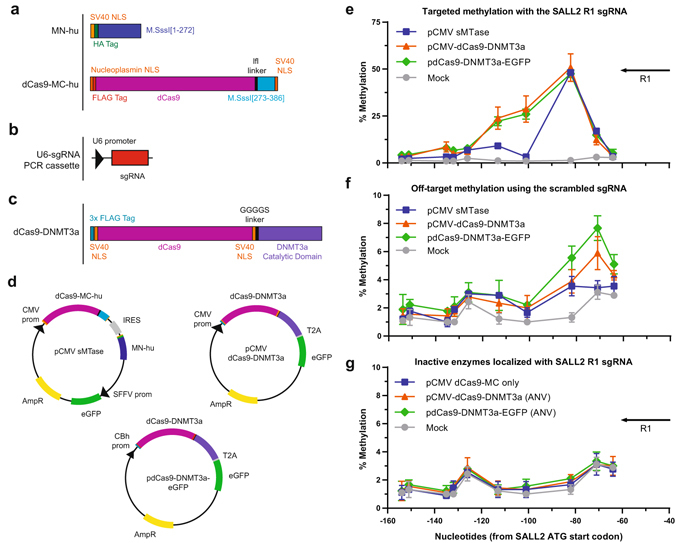
Comparison of the dCas9-sMTase system versus dCas9-DNMT3a fusion. (**a**) Codon optimized versions of the dCas9-MC and MN proteins for use in human cells with nuclear localization signals (NLS) and tags. (**b**) U6-sgRNA PCR cassettes were used for sgRNA expression. **(c)** dCas9-DNMT3a constructs from Vojta *et al*.^27^. **(d)** Schematics showing the pCMV sMTase, pCMV dCas9-DNMT3a-EGFP, and pdCas9-DNMT3a-EGFP expression plasmids. Methylation for biological replicates (n = 3) were measured at the SALL2 P2 promoter for 10 CpG sites ranging from −64 nt to −154 nt upstream of the translation start site in the HEK293T cell. Methylation levels are shown with standard deviations for plasmids expressing active methyltransferases plasmids using **(e)** R1 sgRNA or **(f)** a scrambled sgRNA. **(g)** Inactive versions of the dCas9-sMTase (dCas9-MC-hu only) or the pCMV dCas9-DNMT3a-EGFP and pdCas9-DNMT3a-EGFP systems using catalytically dead DNMT3a versions (ANV) localized using the SALL2 R1 sgRNA were also tested.

Several modifications to our dCas9-MC/MN system were necessary to test targeted DNA methylation in human cells. We codon-optimized the genes for expression in human cells and added nuclear localization signals (NLS) to ensure proper localization to the nucleus (Fig. 4a). We named the new protein dCas9-MC-hu/MN-hu to distinguish it from the *E. coli* version. We cloned the humanized sMTase into a pCMV plasmid containing a fluorescent reporter protein (eGFP) allowing easy isolation of high-expressing sMTase populations of cells by FACS after only 48 hrs, which were then evaluated by pyrosequencing of target promoters (Supplementary Fig. S5a).

We also developed a reporter system to test targeted DNA methylation using cotransfected pCMV sMTase plasmid along with a PCR-derived U6-sgRNA DNA cassette for expression of the sgRNA (Fig. 4b). A linear PCR cassette for sgRNA expression allowed for a cost-effective way to test multiple sgRNAs with minimal cloning steps. We evaluated DNA methylation after 48 hours. This timeframe minimizes the number of cellular divisions and therefore the effects of endogenous mechanisms, such as DNMT1 maintenance methyltransferase, that could cause additional methylation in the region. Such secondary methylation could confound identification of the initial methylation event(s) caused by the targeted sMTase.

**Figure 5:**
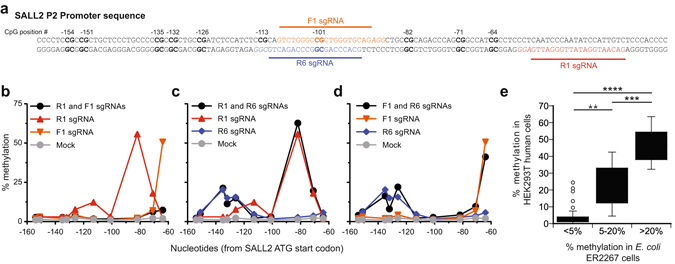
sgRNA design rules for targeted methylation in HEK293T cells. (**a**) Nucleotide sequence relative to the translation start site of the SALL2 P2 promoter showing locations of CpG sites (bold) and sgRNA sequences and PAM sequences (italics). (**b**–**d**) Representative pyrosequencing data showing the percent methylation in the SALL2 promoter in HEK293T cells cotransfected with pCMV-sMTase and either one sgRNA (color coded) or two sgRNAs (black circle). Data shown is from a single representative experiment. Identical trends for all combinations were observed in a replicate experiment. (**e**) Correlation between methylation in HEK293T cells and *E. coli* ER2267 cells at equivalent gap lengths. Percent methylation in *E. coli* as a function of gap length (Fig. 2a) were classified as low (<5%), medium (5–20%) and high (>20%) and the mean and distribution of the corresponding percent methylation values in HEK293T cells were compared by Student’s *t*-test. ****0.0001.

We evaluated dCas9-MC-hu/MN-hu-targeted DNA methylation on a small CpG island in the SALL2 P2 promoter, which regulates the putative tumor suppressor gene SALL2 E1a isoform and is often silenced by hyper-methylation in ovarian cancers^36^. We tested targeted DNA methylation in the easily transfectable HEK293T cell line because this cell line has a hypomethylated SALL2 P2 promoter. We designed pyrosequencing assays to assess DNA methylation on the antisense strand of the SALL2 P2 promoter because the sense strand is T rich after bisulfite conversion making it difficult to pyrosequence this strand. Based on our design rules relating the distance between CpG and PAM sites determined in *E. coli* (Fig. 2a), we designed an sgRNA (R1) to target a CpG site 22 base pairs downstream from a PAM site and located −82 nt from the translation start site (Fig. 5a). We also expected R1 would methylate the CpG site at −71, but at lower levels.

In initial experiments, we compared dCas9-MC-hu/MN-hu containing the (GGGGS)_3_ linker used in the *E. coli* experiments to a longer linker containing a 36-residue negatively-charged flexible linker (lfl), designed to reach CpG sites that were more than 24 bp away from a PAM site **(**Supplementary Fig. S5b **)**. R1 directed both constructs to specifically methylate CpGs at −82 and −71 (Supplementary Fig. S5c). In contrast, a scrambled control sgRNA led to DNA methylation that was only slightly above levels detected in mock transfections or in controls using a vector lacking MN-hu (Supplementary Fig. S5d,e). These initial experiments confirmed that our constructs (1) efficiently methylated a target CpG site within 48 hrs, (2) required localization of the dCas9-MC-hu fragment near the target site; and (3) required expression of both dCas9-MC-hu and MN-hu. Because the construct using lfl resulted in higher levels of DNA methylation at the −82 CpG site (Supplementary Fig. S5c), subsequent experiments used the lfl linker.

### Comparison of dCas9-MC-hu/MN-hu and dCas9-DNMT3a

We next wished to test the precision and specificity of targeted DNA methylation of our bacterial split methyltransferase design. We directly compared DNA methylation of dCas9-MC-hu/MN-hu and previously published dCas9 fusions to the catalytic domain of DNMT3a^23–27^. Vojta *et al*. reported that dCas9-DNMT3a fusions caused high levels of methylation over ~35 bp region centered around a gap length of 27 nt. We tested two plasmids expressing dCas9-DNMT3a: pdCas9-DNMT3a-EGFP (Addgene #71666)^27^ and a version of our plasmid with dCas9-DNMT3a cloned in place of dCas9-MC-hu/MN-hu (Fig. 4c,d). Each plasmid was assessed using the SALL2 R1 sgRNA (test) for on-target and scrambled sgRNAs (control) for off-target DNA methylation (Fig. 4e–g). All three plasmids equally methylated the −82 CpG target site. However, in addition to the −82 CpG site, the dCas9-DNMT3a plasmids also moderately methylated surrounding CpG sites, as predicted by Vojta *et al*., likely due to its ability to recruit additional DNMT3a/DNMT3L complexes that enable spreading of DNA methylation. Using a scrambled control sgRNA, we tested off-target DNA methylation. We observed slightly higher levels of DNA methylation with the dCas9-DNMT3a constructs at certain sites, such as the −71 CpG site. We also tested DNA methylation by inactive MTase controls for each plasmid (pCMV dCas9-MC-hu only and dCas9-DNMT3a-EGFP (ANV) mutant (Addgene #71685)^27^. None of the DNA methylase-inactive controls showed increased DNA methylation. These data demonstrate that we can achieve similarly high levels of DNA methylation with both MTase tools but the sMTase system was more precise in targeted DNA methylation.

### Design rules for multiplexed DNA methylation

The ability to target DNA methylation to multiple CpG sites simultaneously would expand the usefulness of our enzyme. However, dCas9 is a large protein that binds to ~23 base pairs of DNA and is known to cause distortion of the DNA strands^37^. We assessed the rules for placing multiple sgRNAs in close proximity using the sgRNAs R1, F1, and R6 (Fig. 5a). We designed F1 to be 22 bp away from its −64 CpG target site. F1 and R1 bind opposite strands and each target CpG sites within 4 nucleotides of each other’s PAM site. We designed R6 to bind in the same region as F1, but on the opposite strand; thus, R6 and F1 serve as competing sgRNAs. R6’s location was not at an optimal distance away from a CpG site since the primary goal was to create a sgRNA that overlapped F1. However, we expected R6 to cause some DNA methylation at −132 (gap length of 16).

Like R1, F1 and R6 targeted DNA methylation to CpGs when used individually (Fig. 5b–d). However, when R1 and F1 were used together, interference occurred with very little DNA methylation at either target CpG (Fig. 5b **)**. This result indicates that a CpG site that is only 4 bp away from a first sgRNA’s protospacer site cannot be efficiently methylated using a second sgRNA. This result is likely due to steric blocking between the two proteins or due to distortion of the DNA near the target CpG site due to dCas9 binding. Interestingly, we detected low levels of the F1 predicted methylation at CpG site −64 which may indicate different efficiencies of binding between the F1 and R1 sgRNA sequences. In contrast, when R1 and R6 were used together, no interference in DNA methylation at target CpG sites was observed (Fig. 5c), indicating that sgRNAs binding 33 base pairs apart on the same strand can function independently. Surprisingly, F1 and R6 used together were almost as effective as when they were used individually (Fig. 5d), indicating that two sgRNA’s that are designed for the same genomic site but on different strands will function with minimal interference. Because only one dCas9-MC-hu molecule can occupy the Cas9 binding site at a time, the dCas9-MC-hu/sgRNA complex must have a sufficiently fast off-rate from the DNA to allow exchange of the two possible complexes at this site. However, the nearly complete interference exhibited by the F1-R1 combination (Fig. 5b) suggests that protospacer sites targeted by a sgRNA are occupied by a dCas9-MC-hu a majority of the time.

### Multiplexed RNA-programmed DNA methylation in human cells

**Figure 6:**
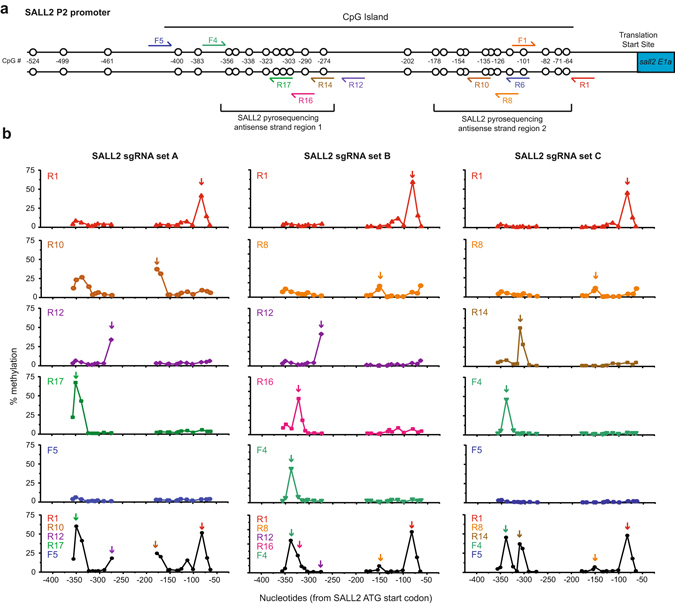
Highly-specific targeting of methylation in the SALL2 P2 promoter using single and multiplexed sgRNAs. (**a**) Schematic of the human SALL2 P2 promoter with sgRNA binding sites and CpG sites (circles) marked by distance from the translation start site of the *sall2 E1A* isoform. (**b**) Representative pyrosequencing data showing the percent methylation in the SALL2 promoter in HEK293T cells cotransfected with pCMV-sMTase and the indicated single or the following multiplexed sgRNAs: SALL2 sgRNA set A (R1, R10, R12, R17 and F5), SALL2 sgRNA set B (R1, R8, R12, R16 and F4), and SALL2 sgRNA set C (R1, R8, R14, F4 and F5). Supplementary Fig. S6 shows overlays of the methylation patterns. The F5 sgRNA targets a site outside the pyrosequencing region but was included to show the lack of off-target methylation at other sites.

Based on our rules for multiplexing sgRNAs, we designed nine additional sgRNAs targeting specific CpG sites at locations spread throughout SALL2 P2 promoter (Fig. 6a). We first analyzed DNA methylation at all CpG sites using each of the sgRNAs separately (Fig. 6b). This constituted 38 different sgRNA-gap distance pairs (most assessed in replicate experiments) and 19 different PAM-CpG gap lengths. We compared HEK293T DNA methylation levels with *E. coli* DNA methylation levels at equivalent PAM-CpG gap distances. In general, we found that rules determining DNA methylation in *E. coli* (Fig. 2a) were predictive of DNA methylation in human cells (Fig. 5e). In particular, no gap lengths leading to high levels of DNA methylation in *E. coli* led to low levels of DNA methylation in human cells. This result is striking when considering all the DNA structural differences between plasmids in *E. coli* and chromosomal DNA in the nucleus of HEK293T cells.

In only one case did a sgRNA cause significant levels of DNA methylation far from its intended site. R10 reproducibly caused DNA methylation at four sites in the −350 and −300 region in addition to DNA methylation at its expected sites of −178 and −170 (Fig. 6b). No PAM-containing sequence with partial complementarity to R10 could be identified near this region. Because our data demonstrate that off-target DNA methylation primarily occurs when dCas9-MC/MN is bound at its intended sgRNA-target site (Fig. 3), the off-target methylation in the −350 and −300 region is likely due to topology of the SALL2 P2 promoter which brings these CpG sites near the target site.

Next, we tested the efficiency of three different sets of five sgRNAs for multiplexed targeted methylation in comparison to single sgRNAs (Fig. 6b and Supplementary Fig. S6). DNA methylation efficiency for multiplexed sgRNAs closely matched individual sgRNAs efficiency (i.e. without interference) (Fig. 6b). The one exception to this observation is the −274 CpG site targeted by R12 is unmethylated in the context of multiplex set B, which can be explained by the fact that R12 violates our multiplexing design rule. The targeted site at −274 is one nucleotide away from the R16 sgRNA protospacer site and is likely obstructed by dCas9 binding.

Pyrosequencing does not provide information on the co-occurrence of DNA methylation at CpG sites on the same molecule of DNA. Instead, pyrosequencing provides information on the frequency of DNA methylation of a sample population (i.e. each site is measured across a population). We used Sanger bisulfite sequencing to test whether multiplexing sgRNAs could direct methylation to the same DNA molecule (Supplementary Fig. S7). These experiments confirmed same-molecule DNA methylation at multiple sites that correspond to sites with high degrees of methylation observed by pyrosequencing. We conclude that dCas9-MC-hu/MN-hu in combination with sgRNAs spaced appropriately along the DNA is capable of directing methylation to multiple target sites in a single promoter region.

The Sanger bisulfite sequencing also provided further evidence supporting our design rules. The SALL2 promoter region near −383 was outside of the region assessed by pyrosequencing analysis preventing us from interrogating the F5 target site. The Sanger sequencing data, however, includes −383 and confirms that F5 causes methylation on the antisense strand of this site (Supplementary Fig. S7). Additionally, the Sanger sequencing data at the −338 and −383 sites shows relatively high antisense strand methylation and little to no methylation on the sense strand (Supplementary Fig. S7). Our design rules correctly predicted the bias for antisense strand methylation at these sites. One exception to our rules is the R8 sgRNA which we predicted to have higher methylation levels at CpG site −154 on the promoter sense strand. While we were able to detect methylation on the antisense strand at low levels, clonal bisulfite sequencing showed no methylation on the sense strand. This could indicate that the −154 site is inaccessible due to chromatic structures or the binding of another protein. Attempts to methylate the nearby −151 CpG site with another sgRNA also showed lower DNA methylation levels than expected (data not shown). These data demonstrate that while our rules accurately predicted targetable CpG sites, other factors can influence the relative amounts of DNA methylation.

### Multiplexing multiple promoters

**Figure 7:**
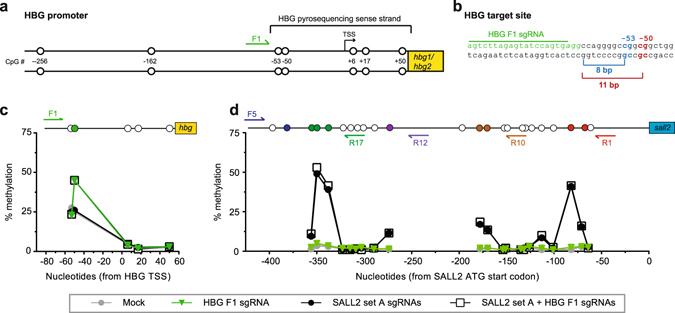
Methylation at multiple promoters using multiplexed sgRNAs. (**a**) Schematic of the HBG (fetal hemoglobin) human promoters with CpG sites (circles) marked by position from the transcription start site (TSS). The relative position of the HBG F1 sgRNA site (green) is shown on the promoter along with (**b**) the detailed nucleotide sequence showing the PAM site in italics. The −50 (red) CpG site is at a good gap distance for methylation whereas the −53 (blue) CpG is not. Representative pyrosequencing data showing the percent methylation in the (**c**) HBG or (**d**) SALL2 promoter in HEK293T cells cotransfected with pCMV-sMTase and the indicated single or multiplexed sgRNAs. The promoter schematics above the graphs indicate the location of sgRNA binding sites and CpG sites (circles). Filled circles indicate the CpG sites at which some level of methylation was expected based on our design rules.

We next tested whether multiplexed sgRNAs could target two different promoters simultaneously without interference or crosstalk (Fig. 7). The human fetal hemoglobin (HBG) promoters contain multiple DNA methylation sites that correlate with silencing of fetal hemoglobin genes, HBG1 and HBG2^38^. The HBG promoters are representative of many promoters that lack CpG islands but instead contain CpG sites spread out over significant distances, which in some cases have a limited number of PAM sites available for designing sgRNAs for targeted DNA methylation. HBG1 and HBG2 are nearly identical so we designed a single sgRNA, HBG F1, to target both simultaneously **(**Fig. 7b **)**. According to our design rules, HBG F1 is expected to cause DNA methylation of the sense strand cytosine at −50 but not −53. We transfected HEK293T cells with the pCMV sMTase plasmid and either the HBG F1 sgRNA alone, the SALL2 sgRNA Set A alone, or both. We assessed DNA methylation by bisulfite sequencing of both HBG promoters **(**Fig. 7c **)** and the SALL2 promoter **(**Fig. 7d **)**. Increased DNA methylation at targeted sites occurred only when the respective promoter’s sgRNAs were present. Multiplexing HBG F1 and the SALL2 Set A sgRNAs to target DNA methylation to both promoters did not alter the efficiency at which each promoter was methylated using their respective sgRNAs (Fig. 7c,d). Our data demonstrate that targeting DNA methylation at one promoter did not affect targeting DNA methylation at a distinct promoter.

## Discussion

We developed a programmable MTase with low off-target effects in human cells. We provide clear design rules governing efficient on-target DNA methylation and multiplexing of sgRNAs for targeting multiple CpG sites without interference. Specifically, we characterized methylation of plasmid DNA in *E. coli* to establish rules that predict how DNA methylation will vary at each cytosine in a CpG site as a function of distance from the PAM. Human genomic DNA is compartmentalized in the nucleus, is bound by histones and other DNA-associated proteins, and is assembled into higher-order chromatin structures that affect DNA topology and CpG site availability. Despite these differences in subcellular localization and organization, the rules determining targeted DNA methylation in bacterial cells were remarkably transferrable when applied to an unmethylated promoter region in human cells. Indeed, the few instances of DNA methylation at unexpected sites were typically low-level methylation at CpG sites proximal to target sites.

Previous studies of targeted DNA methyltransferases^23–27, 39–43^ were characterized solely in mammalian cells, where endogenous CpG methylation complicates assessment of the efficacy and specificity of targeted methylation. The DNMT3a catalytic domain of the dCas9-DNMT3a fusion protein is known to operate as a tetramer and likely recruits additional endogenous DNMT3a/DNMT3L MTases to spread methylation over a larger region^27^. Such effects complicate analysis of the effects of methylation per se versus the effects of localizing DNMT3a to the promoter. In contrast, M.SssI, as a bacterial protein, is not expected to do the same. In addition, previous studies of targeted MTases have not established why off-target sites, sometimes hundreds of bp away, are methylated. The dCas9-DNMT3a methyltransferases are not designed to require dCas9 binding to methylate DNA; thus, dCas9-DNMT3a molecules not bound at their sgRNA-determined site are free to methylate other CpG sites in the promoter or the genome. In contrast, we provide evidence that the predominant source of off-target methylation for our sMTase lies with the ability of dCas9-MC/MN bound at its intended sgRNA-target site to methylate select CpG sites that happen to be located close in space due to DNA topology.

We developed our MTase as a tool to help scientists investigate the mechanisms of DNA methylation initiation, spreading, and inheritance; to discern the relationships between DNA methylation and generation of higher-order chromatin structures; and, to connect the effect of DNA methylation to phenotype. dCas9-MC-hu/MN-hu offers high precision and predictability for placing DNA methylation marks on the DNA with minimal off-target methylation. We believe this specificity arises from its split-enzyme design that links dCas9 binding and MTase activity.

For therapeutic applications, precision in targeting methylation is desirable to avoid unintended effects, provided that a few carefully placed methylations would have a therapeutic benefit. For some promoters, methylating individual CpG sites are important for gene silencing, e.g. BRCA1^44^ and GSTPI^45^. A highly specific targeted MTase might be preferable for such scenarios. If more widespread methylation in a promoter is necessary for a therapeutic effect, multiple sgRNAs are needed for our enzyme to cause methylation throughout a promoter, which may be challenging due to a limited number of available PAMs in a promoter and the gap restrictions on methylation efficiency. The further discovery and engineering of Cas9 variants with different PAM specificities will enable a wider diversity of sMTase systems to address this issue.

## Methods

### General methods, reagents, and bacterial strains

A deactivated version of the *Streptococcus pyogenes* Cas9 gene containing the D10A and H840A mutations (dCas9) was obtained from Addgene (ID: 48657)^35^. *S. pyogenes* sgRNA was also obtained from Addgene (ID: 44251)^20^. *E. coli* K12 ER2267 cells (F′ *proA* ^+^ *B* ^+^ *lacI* ^*q*^ *Δ(lacZ)M15 zzf::mini-Tn*10 (Kan^R^)/*Δ(argF-lacZ)U169 glnV44* e14^−^(McrA^−^) *rfbD1? recA1 relA1? endA1 spoT1? thi-1 Δ(mcrC-mrr)114::IS10)* were obtained from New England Biolabs, Inc (NEB) and were used for all bacterial studies of methylation. Chemically competent ER2267 were prepared in TSS Buffer as described^46^. Electro-competent ER2267 cells were prepared from cells grown to an optical density of 0.5 as described^47^. Other cloning cells lines purchased from NEB include the ElectroMAX DH5α-E electrocompetent cells, and NEB® Turbo and NEB® 10-beta chemically competent cells.

Most enzymes and reagents for gene construction were purchased from NEB, including all restriction enzymes, Epimark DNA polymerase, T4 ligation kit, Taq ligation Kit, T4 Polynucleotide Kinase, Antarctic Phosphatase, Phusion Master Mix, and Exonuclease III. GoTaq DNA polymerase was obtained from Promega. PureLink Gel Extraction Kit was obtained from Thermo Fisher Scientific. Cloning was done using the Gibson Assembly Master Mix prepared according to published protocols^48^ unless otherwise stated. All primers and gBlocks were ordered from Integrated DNA Technologies. Ultrapure agarose was obtained from Thermo Fisher Scientific. General chemicals and antibiotics are obtained from Sigma, unless otherwise specified.

### Bacterial plasmid modifications

The dCas9-M.SssI [273–386] gene and sgRNA, along with its J23100 promoter and terminators, were inserted into the pARC8^3^ using CPEC method^49^ to create pEncode. Two undesired Fsp1 sites in the dCas9 gene were removed by introducing silent mutations by site-directed mutagenesis. Linkers between dCas9 and M.SssI [273–386] were incorporated via Gibson Assembly. The M.SssI [1–272] gene was introduced into pDIMN2^50^ by sticky-end ligation to create pReporter. Plasmids containing unique gap lengths for target site 1 and other modifications to site 1 were created via Gibson Assembly. Sequences of dCas9-MC and MN are provided in Supplementary Text S1.

### Gap library construction

Inverse PCR was used to place the FspI site (5′-TGCGCA-3′) at each position 2–42 bp downstream from the PAM of site 1 in pReporter to create a total of 41 plasmids. Equal volumes of the 41 PCR reactions were pooled together, and the PCR product of the correct size was purified via gel extraction. Purified DNA (100 ng) was phosphorylated and ligated using T4 PNK and T4 Ligation Kits, respectively. The ligation product was purified with DNA Clean and Concentrator-5 (Zymo Research). The ligation products were transformed into NEB DH5α-E. Plasmid DNA was isolated from the colonies recovered from the transformation plate. Cotransformed electro-competent ER2267 cells with this plasmid DNA library (4 ng) and pEncode (8 ng) were plated on plates containing glucose (0.1% w/v), ampicillin (100 μg/mL), and chloramphenicol (50 μg/mL). Cells recovered en masse from the transformation plate were used directly to make glycerol stocks.

### Plasmid methylation in *E. coli*

A single colony of cells containing the desired plasmids was used to inoculate 5–10 mL lysogenic broth containing the standard set of supplements: glucose (0.2% w/v), ampicillin (100 μg/mL), and chloramphenicol (50 μg/mL). The culture was shaken for 16–18 h at standard cell growth conditions: 250 rpm and 37 °C. Glycerol stocks were created with equal volumes of culture and glycerol (50% w/v) and stored at −80 °C.

For the characterization of DNA methylation by restriction enzyme protection assay, 5–10 ml lysogenic broth containing the standard supplements, arabinose (0.0167% w/v), and IPTG (1 mM) was inoculated using 1 µl of frozen cell stocks. Arabinose induced expression of dCas9-MC, and IPTG induced expression of MN. The culture was shaken at 250 rpm at 37 °C for 16–18 hours. Cells were pelleted at 2880× g for 7 minutes, and their plasmids were extracted with Plasmid Miniprep Kit from Qiagen.

For plasmid DNA whose methylation was quantified using MiSeq, 1 µl of a glycerol stock was used to inoculate 5 mL lysogenic broth containing the standard supplements. Cultures were shaken at 250 rpm at 37 °C for 15 hours. From the overnight culture, 1 mL was transferred into a 500 ml shake flask containing 150 mL lysogenic broth and the standard supplements and shaken at 250 rpm at 37 °C. Arabinose (0.0175% w/v) and IPTG (1 mM) were added at an optical density of 0.3. After 4 hours, 5 mL of the culture was centrifuged, and plasmid DNA was purified from the pelleted cells using the Plasmid Miniprep Kit from Qiagen.

### Restriction enzyme protection assay

Plasmid DNA (180 ng) was incubated with 10 units FspI and 20 units SacI-HF in 10 µl Cutsmart Buffer (NEB) at 37 °C for 1.5 hours. SacI-HF was used for plasmid linearization. The digested DNA was loaded into a 1.2% w/v TAE gel containing ethidium bromide, and electrophoresed at 110 Volts for 50 minutes. Band patterns were visualized under UV light and imaged with Carestream Gel Logic 112.

### High-throughput bisulfite sequencing using Illumina MiSeq

Plasmid DNA (5 μg) was linearized using 80 units SacI-HF in 40 µl Cutsmart Buffer for 5 h at 37 °C. DNA was purified with Purelink Gel Extraction Protocol and eluted in TE Buffer to obtain a final concentration of 10–20 ng/μL. DNA (50 μL) was sheared to 300 bp using Diagenode Bioruptor Pico, with cycle conditions of 30 seconds on/30 seconds off for 13 cycles, with vortexing and 10 s centrifugation after every 3 cycles. Size distribution was confirmed with an Agilent 2100 Bioanalyzer and High Sensitivity DNA Kit. DNA end repair and adapter ligation was performed with NEBNext Ultra DNA library Prep Kit from NEB. Fragments were size selected using Agencourt AMPure XP Beads (0.45X, 0.25X to select 300 bp inserts). DNA recovered with 24 μL nuclease-free water was bisulfite treated with Methylation Lightning Conversion Kit from Zymo Research according to the manufacturer’s instructions. Products were amplified with Kapa Hifi Uracil + ReadyMix from Kapa Biosystems and NEBNext Multiplex Oligos for Illumina (Methylated Adaptor) from NEB; the cycling was performed as followed: initial denaturation 98 °C for 45 sec; 8 cycles of 98 °C for 15 sec, 65 °C for 30 sec, 72 °C for 30 sec; and a final 1 min extension at 72 °C. Samples were purified with AMPure (1X). DNA concentration was determined using qPCR (Kapa Illumina Library Quantification Kit), and size distribution was confirmed using the High Sensitivity Bioanalyzer. Standard Illumina product procedures were used in the preparation of MiSeq with DNA library (4 nM) and MiSeq Reagent Kit v3 2 × 75 cycle. Data was deposited in SRA under Bioproject PRJNA393629.

### Analysis of MiSeq data

MiSeq bisulfite sequencing data was aligned to a reference sequence for the plasmid using bowtie2 via Bismark^51^. After alignment, the average methylation levels were extracted for each cytosine in the reference. A custom R script was then used to plot strand and context specific methylation to compare between samples. Analysis code is provided at https://github.com/timp0/xiong_splitcas9.

### Structural models

A model of a double stranded DNA bound by Cas9/sgRNA complex and M.HhaI was created using Pymol from structures of DNA-bound *S. pyogenes* Cas9 (4UN3) and M.HhaI (2HR1). These structures were superimposed on a model of double stranded B-DNA template consisting of a variable length region between the PAM site and the target CpG site that was built with Model.it web server^52^ (Supplementary Table S2).

### Polar plot analysis of methylation frequency

The angle formed between the C-terminus of Cas9 and N-terminus of M.SssI [273–386] when looking down the DNA axis was determined for all gap lengths ranging from 2 bp to 27 bp. For a gap length of 8 bp, the angle was determined from the model to be 20° for the MTase methylating the trans strand and 290° for the MTase methylating the cis strand. Angles for other gap lengths were calculated assuming ideal B-DNA properties (i.e. a change of 34.3° per added or subtracted bp in the gap length).

### Mammalian expression vector construction

M.SssI fragments and a catalytically dead SPCas9 were codon optimized for mammalian expression using Gene Designer2.0 software^53^. Fragments for the M.SssI[273–386] (MC) with linker, M.SssI [1–272] (MN) and dCas9 fragments were synthesized as gblocks (Integrated DNA Technologies) and assembled into a newly designed pCMV plasmid by Gibson isothermal assembly. An alternative long flexible link sequence was inserted in a subsequent cloning step (See Supplemental Text S2 for the humanized dCas9-lfl-MC-IRES-MN DNA sequence) and both were transformed into NEB 10-beta cell strain. Plasmids with only the optimized dCas9-MC-hu, dCas9-MC-hu and MN-hu under direct control of the CMV promoter were also created and transformed into NEB Turbo cells. These plasmids are available from Addgene as #89930 (pCMV sMTase), #89931 (pCMV dCas9-MC-hu), and #89932 (pCMV MN-hu).

Vectors pdCas9-DNMT3A-EGFP (Addgene plasmid #71666) and pdCas9-DNMT3A-EGFP (ANV) (Addgene plasmid #71685) were a gift from Vlatka Zoldoš. The dCas9-DNMT3a-T2A-eGFP constructs were cloned using PCR from the provided plasmids and transferred into the pCMV plasmid under control of the CMV promoter by Gibson isothermal assembly. This cloning step also removed the SFFV eGFP that is normally present.

### Selection of sgRNA sequences and creation of U6-sgRNA cassettes

HBG and SALL2 guide sequences were designed using sequenced promoter regions from the HEK293T cells (see Supplemental Text S3). When possible, sgRNAs were selected to be 20 nt long and to be at or close to permissible distances from a CpG site on either the sense or antisense strands as determined by E. coli gap length evaluation (See Supplementary Table S3 for guide strand sequences). A scrambled control was cloned using a sequence designed to bind to only 3 sites on genome (GE Dharmacon). PCR cassettes were then created by using primers that amplify a U6 promoter fused to guide sequence and guide sequence fused to remaining sgRNA cassettes, followed by another round of overlap-extension PCR to amplify the full U6-sgRNA cassette following a procedure similar to that found in Ran *et al*.^54^ (See Supplementary Text S4 for sequence).

### Cell culture, transfection and flow cytometry for methylation assays in human cell lines

All experiments were performed in Human embryonic kidney 293 T (HEK293T) cells gifted by Ronny Drapkin. Cells were cultured in DMEM supplemented with L-glutamine (Thermo Fisher Scientific) and 10% FBS and incubated in a humidified incubator at 37 °C and 5% CO_2_. Plasmids and U6-sgRNA cassettes were transfected into HEK293T cells in 6 well plates using Optifect™ Reagent (Thermo Fisher Scientific) according to manufacturer’s instructions when cells were ~40–60%. Typically one well per plasmid/sgRNA condition is used in an experiment. Total DNA transfected was limited to 3–3.5 ug for plasmids and 100 ng for each U6 sgRNA cassette (up to 600 ng total) with 13–14 ul of Optifect™ reagent. Reagent was mixed and transfected into OptiMEM media (Thermo Fisher Scientific) without FBS and incubated for ~4–6 hours to increase transfection efficiency before supplementing FBS to 5%. Cells were recovered ~48 ± 2 hrs after transfection and sorted using a Sony SH800Z flow cytometer. Propidium iodide (PI) viability stain was added (to 1 ug/ml concentration) before sorting and cells were sorted for PI negative (viable) and GFP + cells. Attempts are made to control for variations in transfection efficiencies by setting a minimum GFP fluorescent gates and keeping flow cytometer settings constant when possible.

### Bisulfite sequencing and methylation analysis

Each methylation experiment was repeated (n = 3) to verify trends. Cells were lysed and genomic DNA modified and recovered using the Epitect Fast LyseAll Bisulfite Kit (Qiagen). Bisulfite converted DNA was then PCR amplified using the Epimark® Hot Start Taq DNA polymerase kit (NEB) according to manufacturer’s instructions (annealing temperatures vary) using 20–40 ng of genomic DNA. All primers and pyrosequencing assays were designed in the Pyromark Assay Design 2.0 software and analyzed on the Pyromark Q24 Advanced (Qiagen) using manufacturers recommended protocols. The HBG sense strand was amplified using B1- HBG-sense-rev and HBG-sense-for. Pyrosequencing was done using two sequencing primers, HBG-sense(1)-Pseq and HBG-sense(2)-Pseq, using the same PCR amplicon. SALL2 P2 assays of the anti-sense promoter strand were designed for the two regions of the CpG island. Region one is amplified using primers SALL2-antisense-reg1-for and B8-SALL2-antisense-reg1-rev and sequenced using SALL2-antisense-reg1-Pseq. SALL2 region 2 was amplified with B11-SALL2-antisense-reg2-rev and either SALL2-antisense-reg2-for or SALL2-antisense-reg2–2-for primers and was sequenced using SALL2-antisense-reg2-Pseq.

Clonal bisulfite sequencing was done using samples previously evaluated by pyrosequencing. The SALL2 antisense region 1 was amplified with SALL2-reg1-KpnI-rev and SALL2-reg1-SphI-for and region 2 was amplified with primers SALL2-reg2-KpnI-rev and SALL2-reg2-SphI-for. For the sense strand, a set of nested primers were used with SALL2-sense-for and SALL2-sense-rev used in first step PCR followed purification of the product. The second PCR was done using 1 ng of first sense PCR as a template and primers SALL2-sense-KpnI-for and SALL2-sense-SphI-rev. Final PCR amplicons were gel purified and digested with SphI-HF and KpnI-HF restriction enzymes and ligated into a similarly digested and dephosphorylated pUC19 vector. Ligated DNA was transfected into Turbo cells and colonies sequenced by Sanger sequencing (Genewiz). All bisulfite sequencing primer sequences are listed in Supplementary Table S4.

## Electronic supplementary material

Supplementary Information
