# Supplementary material for: Targeted DNA methylation in human cells using engineered dCas9-methyltransferases
Source: Sci Rep. 2017 Jul 27;7:6732. doi: 10.1038/s41598-017-06757-0 (PMC5532369; doi:10.1038/s41598-017-06757-0)
Supplement: Supplementary file 1 — Supplementary Information [file 41598_2017_6757_MOESM1_ESM.pdf]

Supplemental Information

Targeted DNA methylation in human cells using engineered dCas9-methyltransferases

Tina Xiong[1*], Glenna E. Meister[2,3,4*], Rachael E. Workman[5], Nathaniel C. Kato[1], Michael J. Spellberg[2,3,4], Fulya Turker[2,3,4,6], Winston Timp[5], Marc Ostermeier[1†], and Carl D. Novina[2,3,4†]

[1]Department of Chemical and Biomolecular Engineering, Johns Hopkins University, Baltimore, MD, 21218. [2]Department of Cancer Immunology and Virology, Dana-Farber Cancer Institute, [3]Department of Medicine, Harvard Medical School, Boston, MA 02115. [4]Broad Institute of Harvard and MIT, Cambridge, MA 02141. [5]Department of Biomedical Engineering, Johns Hopkins University, Baltimore, MD, 21218. [6]Boston University, Boston, MA 02215.

*contributed equally
†corresponding authors: oster@jhu.edu, carl_novina@dfci.harvard.edu

Supplementary Figures S1-S7
Supplementary Tables S1-S4
Supplementary Text S1-S4

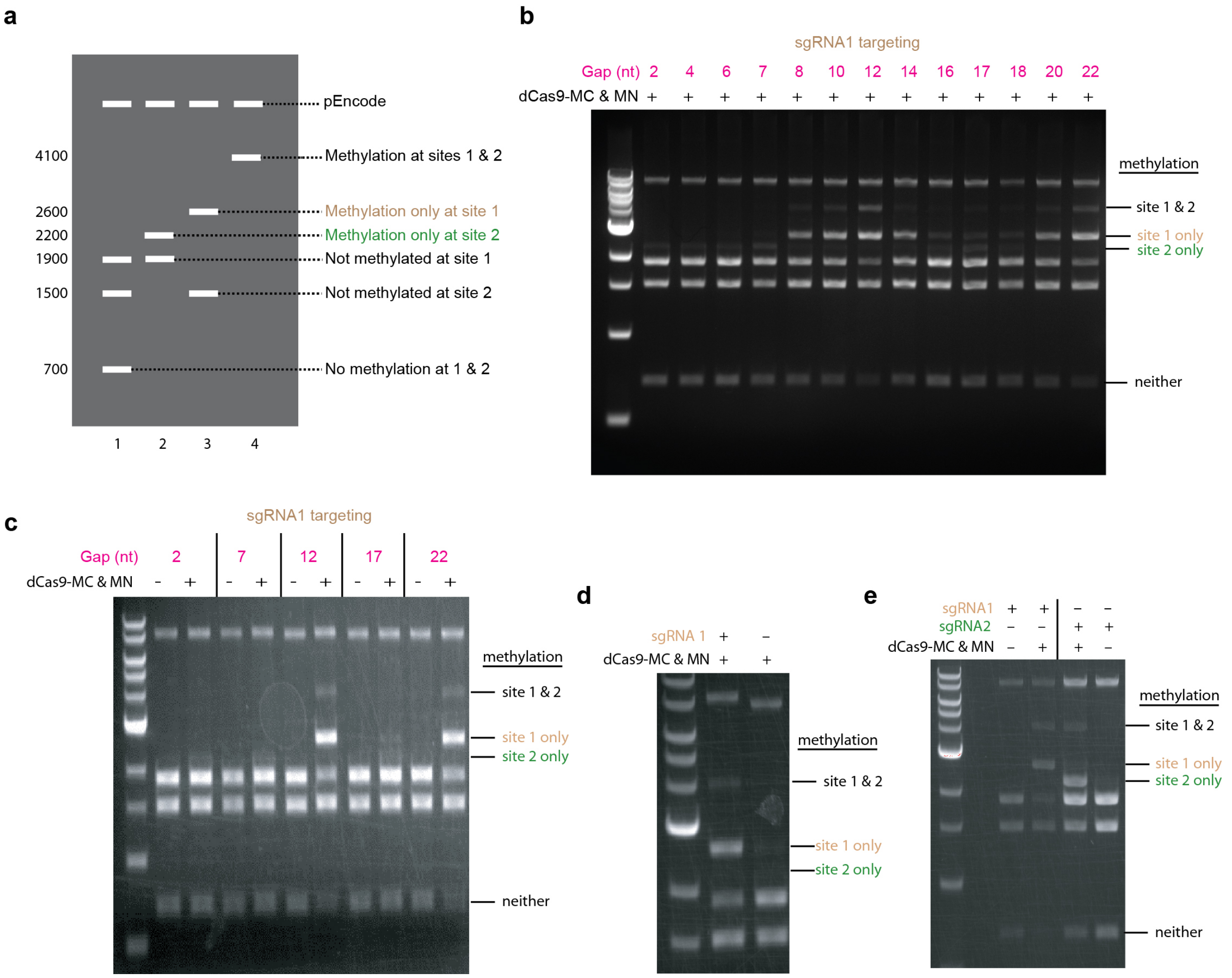

Supplementary Figure S1. Targeted methylation assessed by protection from restriction enzyme digestion. (**a**) Mock gel of the restriction digest protection assay. Gel shows the expected results of FspI digestion of pReporter as a function of the methylation state of sites 1 and 2. Lane 1, neither site methylated; lane 2, only site 1 methylated; lane 3, only site 2 methylated; lane 4, both sites 1 and 2 methylated. Diagnostic bands for these four possibilities are indicated to the right. Plasmid pEncode has no FspI sites and is not digested. (**b**) Methylation pattern encoded by sgRNA1 as a function of the gap between the PAM site and the CpG site when dCas9-MC and MN are co-expressed. sgRNA1 targets methylation to site 1. (**c**) Targeted methylation requires induction of expression of dCas9-MC/MN (–, no induction; +, induction of both dCas9-MC and MN). (**d**) Targeted methylation requires the sgRNA. In lane 2 the sgRNA gene and its promoter were deleted from pEncode. (**e**) The sgRNA encodes the targeting of methylation.

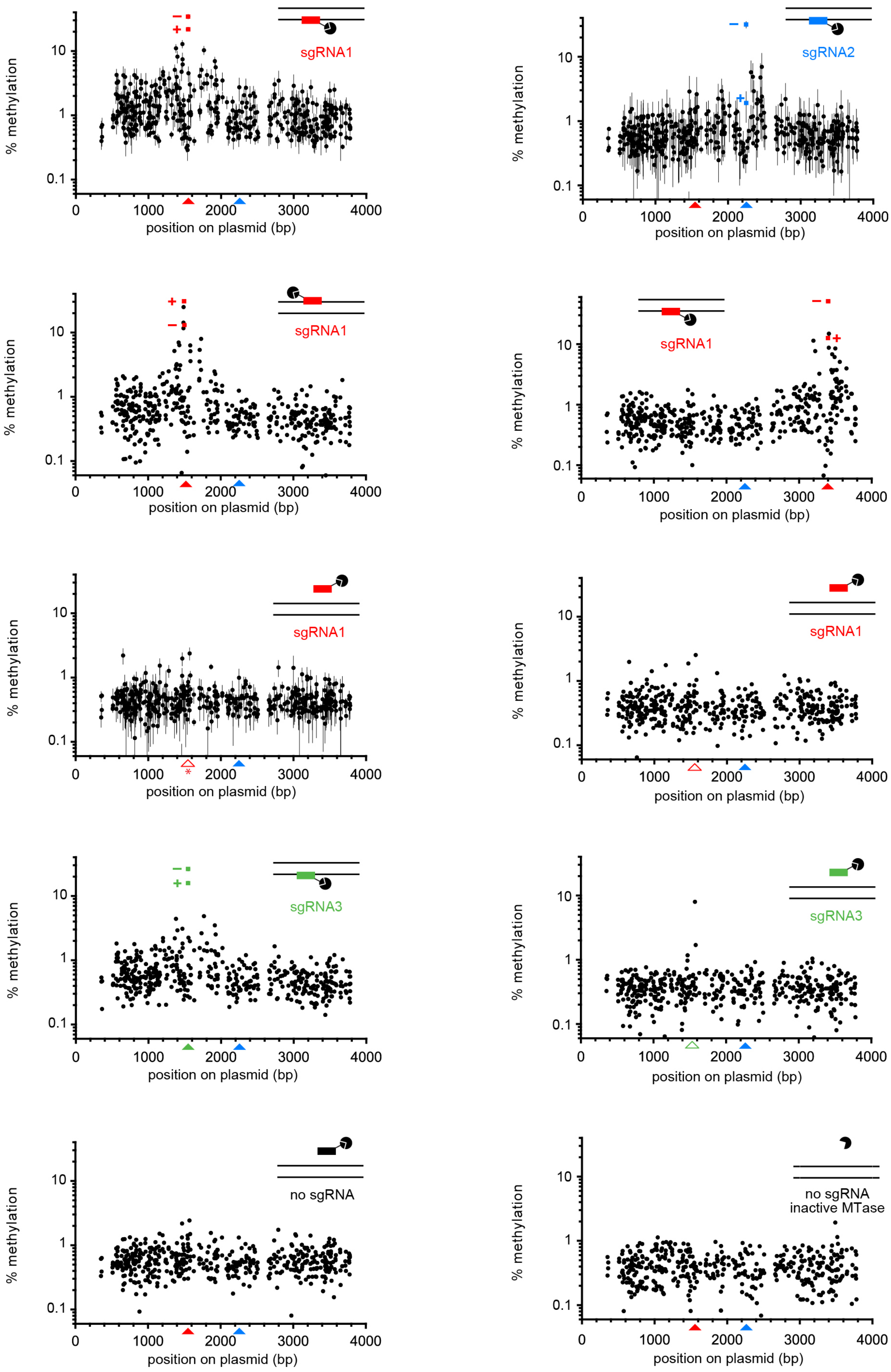

% methylation
position on plasmid (bp)
0
1000
2000
3000
4000
0.1
1
10
sgRNA1
sgRNA2
sgRNA1
sgRNA1
sgRNA1
sgRNA1
sgRNA3
sgRNA3
no sgRNA
no sgRNA
inactive MTase

Supplementary Figure S2. Fraction methylated of the 484 cytosines in 282 CpG sites in pReporter as a function of position on the plasmid, sgRNA, and protospacers. The fraction methylated of target sites CpGs (colored squares) and the 482 off-target sites (black circles) is shown. In each experiment, the sgRNA is indicated as well as whether the target DNA is on the top strand (+), the bottom strand (−), or no strand since the plasmid lacks a protospacer matching the sgRNA. The presence and location of the sgRNAs' target sites on the plasmid are indicated by a solid matching color triangle below the x-axis. An open triangle indicates a scrambled protospacer. An asterisk indicates that the PAM site also is scrambled. Error bars in indicate the standard deviation (n=3 independent cultures). Experiments without error bars were performed once.

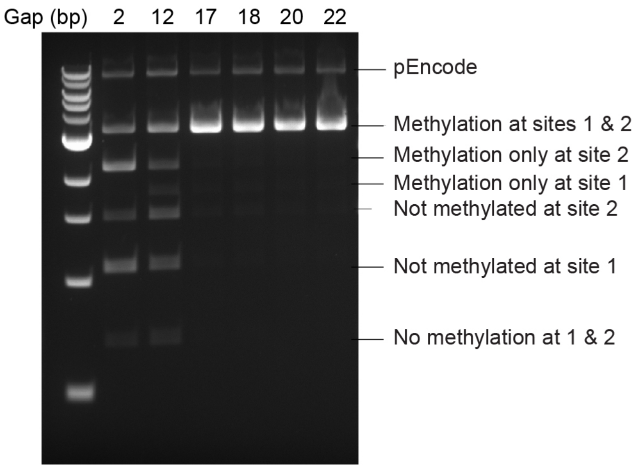

Supplementary Figure S3. Restriction enzyme protection assay of plasmid DNA from cells expressing dCas9-M.SssI[1-386]. Plasmid DNA was analyzed from cells expressing dCas9-M.SssI[1-386] (i.e. an end-to-end fusion of dCas9 and an unfragmented M.SssI) and sgRNA1, which targets site 1. Cells contained different versions of pReporter with the indicated gap lengths between the PAM and the target CpG site.

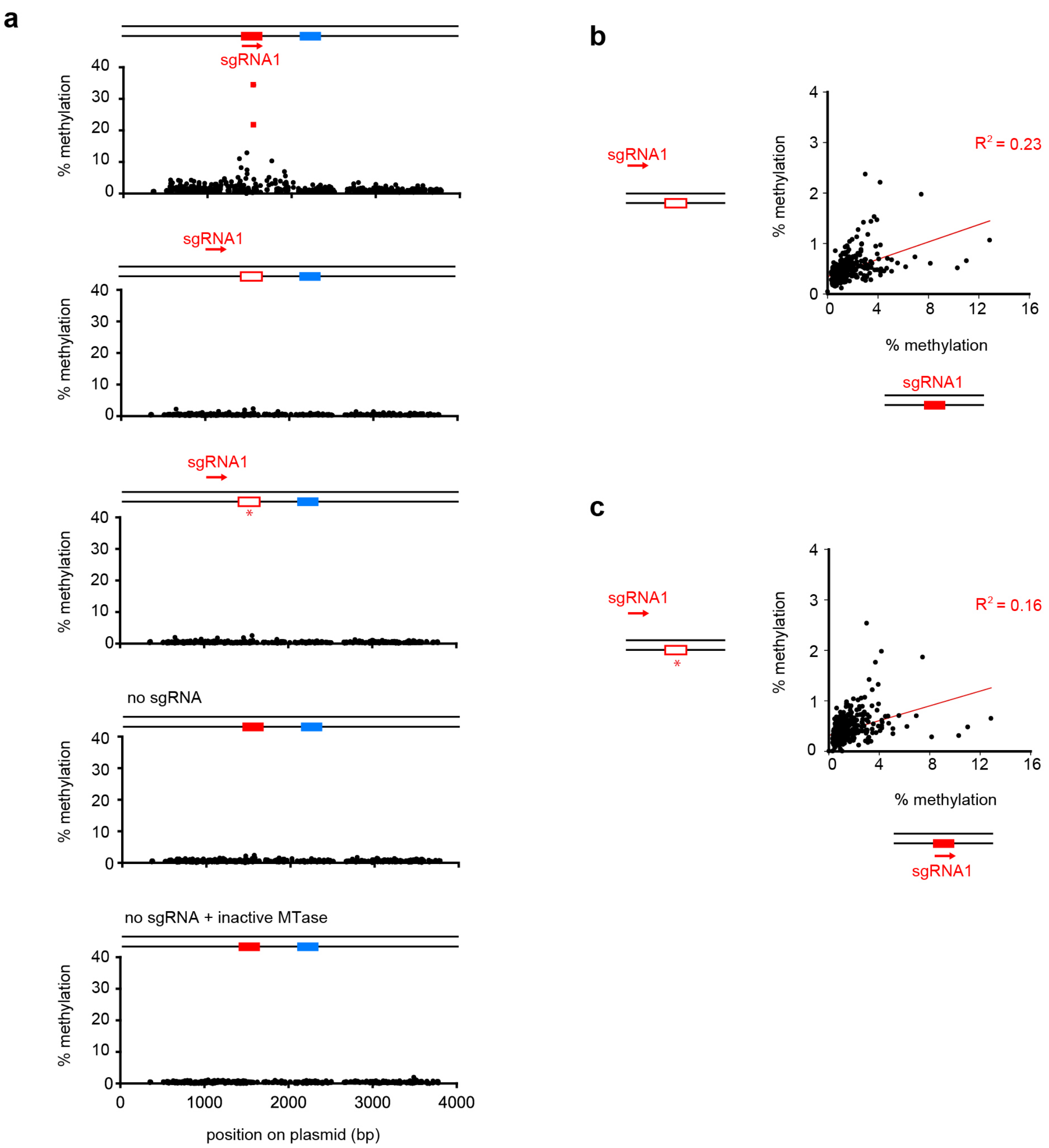

Supplementary Figure S4. The role of the protospacer in off-target methylation. Removing sgRNA1's protospacer from the plasmid decreased the mean level of sgRNA1-guided methylation at off-target sites and changes the pattern of methylation. (**a**) The frequency of methylation at target sites CpG (colored squares) and the 482 off-target sites (black circles) is shown. In each experiment, the sgRNA used is indicated. The presence and location of an sgRNA's binding site on the plasmid is indicated by a colored rectangle on the schematic of dsDNA above the graph the x-axis. An open rectangle indicates a scrambled site. An asterisk indicates that the PAM site also is scrambled. (**b**) Scrambling sgRNA1's protospacer or (**c**) scrambling both the protospacer and the PAM site on the DNA changes the pattern of methylation level at off-target site.

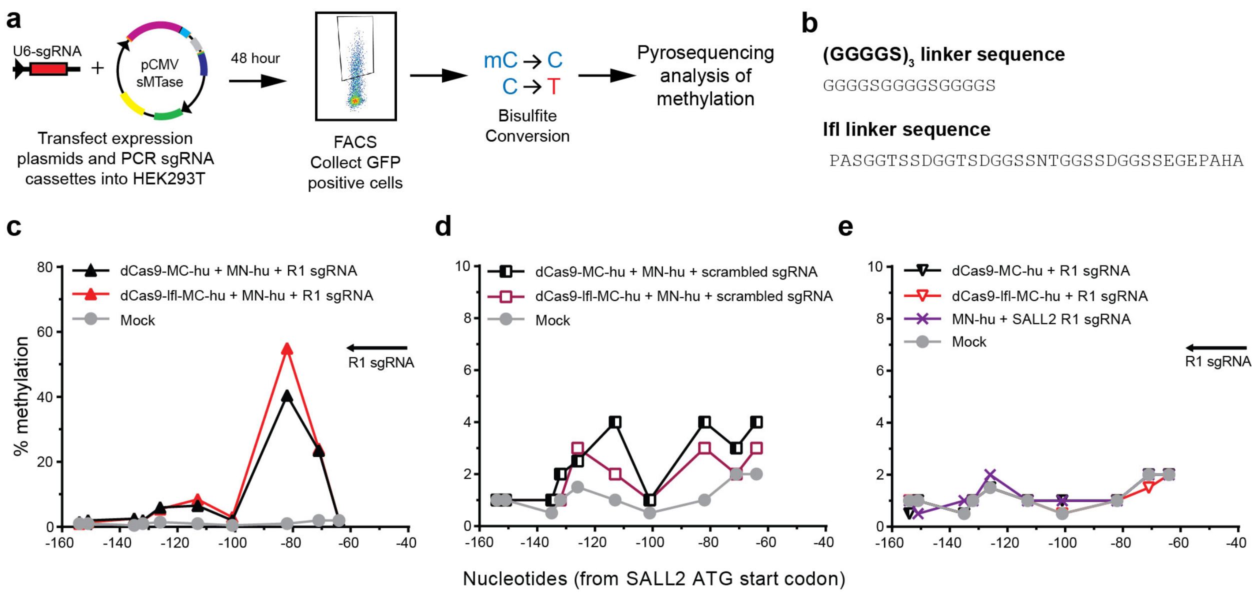

Supplementary Figure S5. Optimized assay and construct in mammalian cells allow for high levels of on-target with minimal off-target DNA methylation. (**a**) Assay schematic using a U6-sgRNA pcr cassette and the pCMV sMTase expression plasmid coexpressing the MN-hu and dCas9-MC-hu with either the $(GGGGS)_3$ or lfl linker [sequences shown in (**b**)]for detection of methylation activity in cell lines. Pyrosequencing data of a representative experiment comparing methylation at the SALL2 P2 for cells cotransfected with pCMV sMTase plasmids and either (**c**) R1 sgRNA, or (**d**) scrambled control sgRNA. (**e**) Inactive methyltransferase controls for plasmid containing only one sMTase fragment (but including R1 sgRNA). Mock transfection controls (grey circles) are shown in all graphs for comparison.

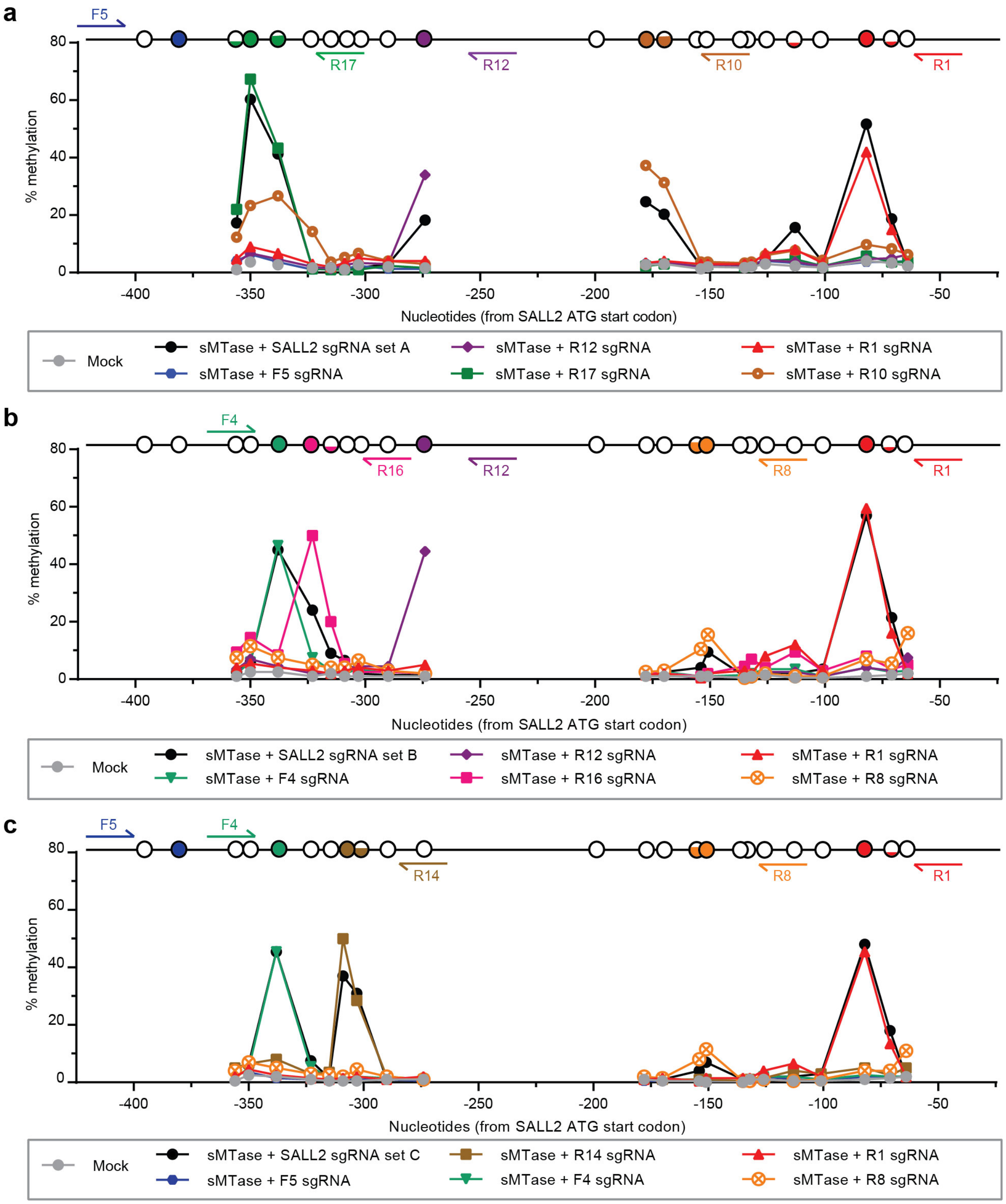

Supplementary Figure S6. Overlay of single versus multiplexed sgRNA methylation amounts for SALL2 sets A, B and C. Methylation amounts for a single (color coded) and multiplexed (black circles) SALL2 sgRNA sets. Diagrams showing the methylation sites are shown above graphs of a representative experiment for (**a**) SALL2 sgRNA set A (R1, R10, R12, R17, F5), (**b**) SALL2 sgRNA set B (R1, R8, R12, R16, F4), and (**c**) SALL2 sgRNA set C (R1, R8, R14, F4, F5) plots. Fully-colored circles show the CpG sites with predicted highest methylation for each individual guide strand with observed lower methylation secondary and tertiary sites shown as partially colored.

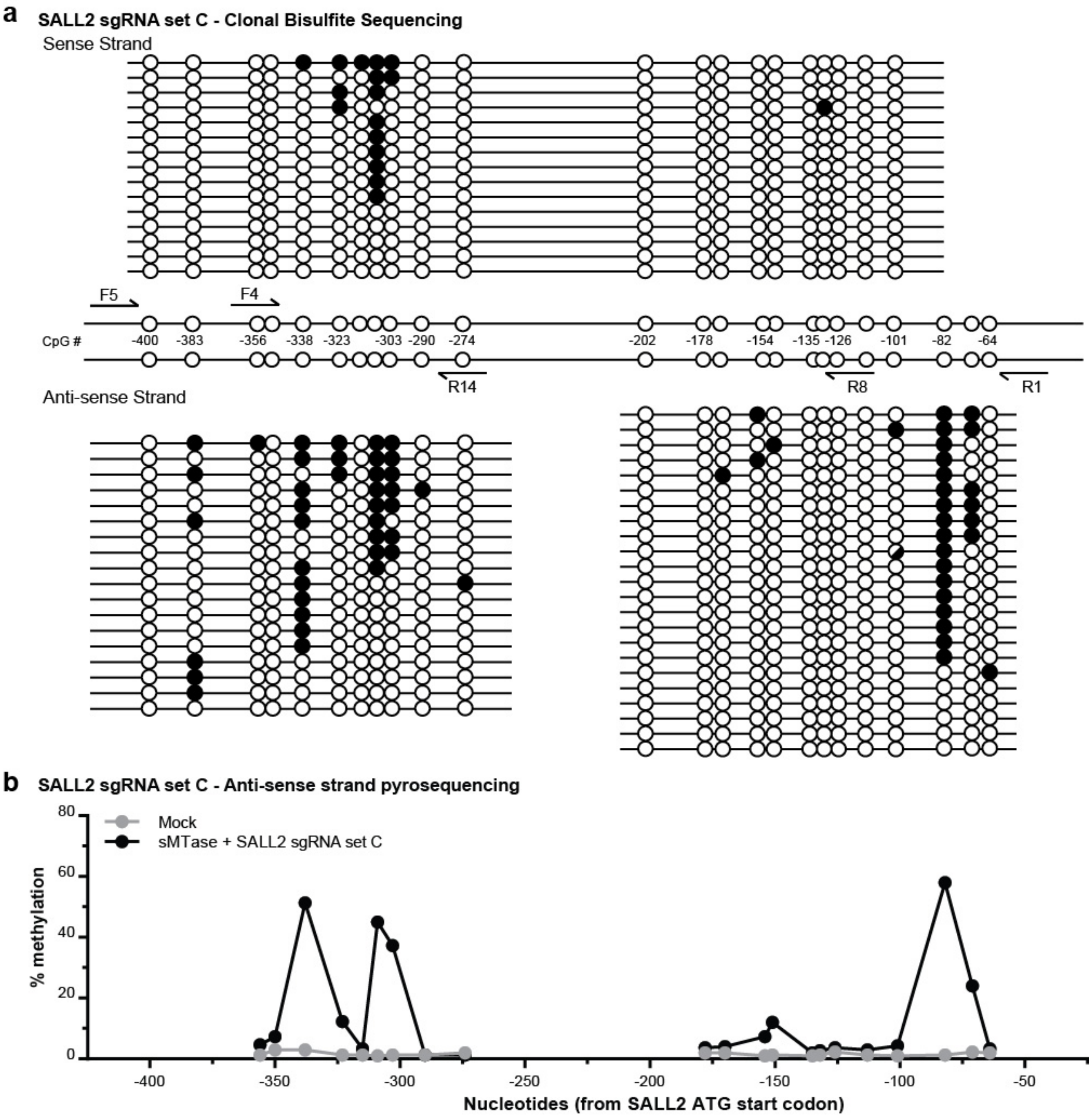

Supplementary Figure S7. Clonal bisulfite sequencing of the SALL2 P2 promoter strands shows multiple sites can be methylated in a single promoter using multiplexed sgRNAs. (**a**) Clonal bisulfite sequencing for both sense (top) and antisense (bottom) strands in a single population of HEK293T cells transfected with the pCMV sMTase plasmid and the SALL2 set C sgRNAs. Locations of sgRNAs are shown on a diagram of the CpG sites in this region of the promoter. (**b**) Pyrosequencing of the antisense strand for the same regions.

Supplementary Table S1. Sequence of target region consisting of dCas9 binding site, CpG site and overlapping FspI site.

Site	DNA sequence[a]
Site 1	5'-AACTTTCAGTTTAGCGGTCT**AGG**CTTACGCTAATGCGCA-3'
Site 2	5'-CACCACGATGCCTGTAGCAA**TGG**CAACAACGTTGCGCA-3'
Site 1 (scrambled)	5'- ATTAGCTGTGCCTATTAGTC**AGG**CTTACGCTAATGCGCA -3'
Site 1 (scrambled; no PAM)	5'-GTTGCCGCTTAGCTATAGTAGATCTTACGCTAATGCGCA-3'
Site 3	5'- ATTAGCTGTGCCTATTAGTC**AGG**CTTACGCTAATGCGCA -3'
Site 3 (scrambled)	5'- AACTTTCAGTTTAGCGGTCT**AGG**CTTACGCTAATGCGCA -3'

[a]In bold is the PAM sequence. Red corresponds with the cytosine that is targeted for methylation. This cytosine has an overlapping FspI site ('TGCGCA').

Supplementary Table S2. Sequences of alignment templates created with Model.it.

Gap (bp)	DNA sequence[a]
10	5'-A**TGG**TATT*TGATA*GCGCTATC-3'
12	5'-A**TGG**TATTTTA*GATA*GCGCTATC-3'
17	5'-A**TGG**TATTCGATATA*TGATA*GCGCTATC-3'

[a]In bold is the PAM sequence. Red corresponds with the cytosine that is targeted for methylation. Underlined region and italicized region are regions that are aligned with DNA sequences from 4UN3 and 2HR1, respectively.

Supplementary Table S3. Sequences of human promoter sgRNA target regions

Guide Name	Promoter	Guide Sequence (5' to 3'
Scrambled	none	GTCGTCTACATGTACGACTA
HBG F1	HBG1/HBG2	GAGTCTTAGAGTATCCAGTG
F1	SALL2 P2	GTCTGGGCCGCTGGGTGCAG
F4	SALL2 P2	AGCACCTGTCACTGCGCCTC
F5	SALL2 P3	GCAGATGGGGGGAGCTGGCC
R1	SALL2 P2	GACAATGGATATTGGGATTG
R6	SALL2 P2	GCACCCAGCGGCCCAGACTG
R8	SALL2 P2	GCGGAGATGGAGATCGGCAG
R10	SALL2 P2	GCGGGGGCAGGGAGCAGCGG
R12	SALL2 P2	TGATGAGGAGGGGAGTTTAT
R14	SALL2 P2	GGGAGGGAGGCGGGAGCTAG
R16	SALL2 P2	GAGCTAGAGGAGGCGGGAGA
R17	SALL2 P2	GCTAGCGGGGGCGTGGGGGC

Supplementary Table S4. Bisulfite sequencing primers for methylation analysis

**Name**	**Sequence (5'-3')**
**Biotinylated primers for pyrosequencing**	
B1 - HBG-sense-rev	BIOTIN-CAAACTTATAATAATAACCTTATCCTCCTCTA
B8 - SALL2-antisense-reg1-rev	BIOTIN-CCCTCAATACTTCACAACACCTATCACTA
B11 - SALL2-antisense-reg2-rev	BIOTIN-CCAACAACTCCTCCCCATA
**Amplicon primers for pyrosequencing**	
HBG-sense-for	AGGGAAGAATAAATTAGAGAAAAATTGG
SALL2-antisense-reg1-for	GGGAGAAGTTGGAGTGAGAAA
SALL2-antisense-reg2-for	GGTGGGGTGGGAGATAAT
SALL2-antisense-reg2-2-for	GGAAGTGGAGGGTTAGGT
**Sequencing primers for pyrosequencing**	
HBG-sense(1)-Pseq	TTGATTAATAGTTTTAGAGTATTTAG
HBG-sense(2)-Pseq	TTAGGGATGAAGAATAAAAGG
SALL2-antisense-reg1-Pseq	TATGGGGAGGAGTTGT
SALL2-antisense-reg2-Pseq	TTAGGGATGAAGAATAAAAGG
**Clonal bisulfite sequencing primers**	
SALL2-sense-for	GTAGGTTTGGGTAAAGGTA
SALL2-sense-rev	CTAAATCTACCACAACCTCTACACC
SALL2-sense-KpnI-for	CAGTAGGTACCTGGGTAAAGGTATTTGTAGATGG
SALL2-sense-SphI-rev	ATGTAGCATGCCCTACCACAACCTCTACACCCAAC
SALL2-reg1-KpnI-rev	CAGTAGGTACCCCCACTATACCCCTAAAACTTAA
SALL2-reg1-SphI-for	ATGTAGCATGCGGGAGAAGTTGGAGTGAGAAA
SALL2-reg2-KpnI-rev	CAGTAGGTACCCCAACAACTCCTCCCCATA
SALL2-reg2-SphI-for	ATGTAGCATGCGGTGGGGTGGGAGATAAT

Supplementary Text S1. DNA Sequences of dCas9-MC (15 amino acid linker) and MN for E. coli expression

KEY: dCas9 (purple) and MC (cyan) are joined by a $(GGGGS)_3$ linker (brown). Silent mutations (gray shading) were created to remove FspI sites. J23100 promoter (green) is upstream of the sgRNA1 (orange). Terminator sequences are in red.

< dCas9-MC + sgRNA1>
ATGGATAAGAAATACTCAATAGGCTTAGCTATCGGCACAAATAGCGTCGGATGGGCGGTGATCA
CTGATGAATATAAGGTTCCGTCTAAAAAGTTCAAGGTTCTGGGAAATACAGACCGCCACAGTAT
CAAAAAAAATCTTATAGGGGCTCTTTTATTTGACAGTGGAGAGACAGCGGAAGCGACTCGTCTC
AAACGGACAGCTCGTAGAAGGTATACACGTCGGAAGAATCGTATTTGTTATCTACAGGAGATTT
TTTCAAATGAGATGGCGAAAGTAGATGATAGTTTCTTTCATCGACTTGAAGAGTCTTTTTTGGT
GGAAGAAGACAAGAAGCATGAACGTCATCCTATTTTTGGAAATATAGTAGATGAAGTTGCTTAT
CATGAGAAATATCCAACTATCTATCATCTGCGAAAAAAATTGGTAGATTCTACTGATAAAGCGG
ATTTGCGCTTAATCTATTTGGCCTTAGCGCATATGATTAAGTTTCGTGGTCATTTTTTGATTGAG
GGAGATTTAAATCCTGATAATAGTGATGTGGACAAACTATTTATCCAGTTGGTACAAACCTACA
ATCAATTATTTGAAGAAAACCCTATTAACGCAAGTGGAGTAGATGCTAAAGCGATTCTTTCTGC
ACGATTGAGTAAATCAAGACGATTAGAAAATCTCATTGCTCAGCTCCCCGGTGAGAAGAAAAAT
GGCTTATTTGGGAATCTCATTGCTTTGTCATTGGGTTTGACCCCTAATTTTAAATCAAATTTTGA
TTTGGCAGAAGATGCTAAATTACAGCTTTCAAAAGATACTTACGATGATGATTTAGATAATTTA
TTGGCGCAAATTGGAGATCAATATGCTGATTTGTTTTTGGCAGCTAAGAATTTATCAGATGCTA
TTTTACTTTCAGATATCCTAAGAGTAAATACTGAAATAACTAAGGCTCCCCTATCAGCTTCAATG
ATTAAACGCTACGATGAACATCATCAAGACTTGACTCTTTTAAAAGCTTTAGTTCGACAACAACT
TCCAGAAAAGTATAAAGAAATCTTTTTTGATCAATCAAAAAACGGATATGCAGGTTATATTGAT
GGGGGAGCTAGCCAAGAAGAATTTTATAAATTTATCAAACCAATTTTAGAAAAAATGGATGGTA
CTGAGGAATTATTGGTGAAACTAAATCGTGAAGATTTGCTACGCAAGCAACGGACCTTTGACAA
CGGCTCTATTCCCCATCAAATTCACTTGGGTGAGCTGCATGCTATTTTGAGAAGACAAGAAGACT
TTTATCCATTTTTAAAAGACAATCGTGAGAAGATTGAAAAAATCTTGACTTTTCGAATTCCTTA
TTATGTTGGTCCATTGGCGCGTGGCAATAGTCGTTTTGCATGGATGACTCGGAAGTCTGAAGAA
ACAATTACCCCATGGAATTTTGAAGAAGTTGTCGATAAAGGTGCTTCAGCTCAATCATTTATTG
AACGCATGACAAACTTTGATAAAAATCTTCCAAATGAAAAAGTACTACCAAAACATAGTTTGCT
TTATGAGTATTTTACGGTTTATAACGAATTGACAAAGGTCAAATATGTTACTGAAGGAATGCGA
AAACCAGCATTTCTTTCAGGTGAACAGAAGAAAGCCATTGTTGATTTACTCTTCAAAACAAATC
GAAAAGTAACCGTTAAGCAATTAAAAGAAGATTATTTCAAAAAAATAGAATGTTTTGATAGTGT
TGAAATTTCAGGAGTTGAAGATAGATTTAATGCTTCATTAGGTACCTACCATGATTTGCTAAAA
ATTATTAAAGATAAAGATTTTTTGGATAATGAAGAAAATGAAGATATCTTAGAGGATATTGTTT
TAACATTGACCTTATTTGAAGATAGGGAGATGATTGAGGAAAGACTTAAAACATATGCTCACCT
CTTTGATGATAAGGTGATGAAACAGCTTAAACGTCGCCGTTATACTGGTTGGGGACGTTTGTCTC
GAAAATTGATTAATGGTATTAGGGATAAGCAATCTGGCAAAACAATATTAGATTTTTTGAAATC
AGATGGTTTTGCCAATCGCAATTTTATGCAGCTGATCCATGATGATAGTTTGACATTTAAAGAA
GACATTCAAAAAGCACAAGTGTCTGGACAAGGCGATAGTTTACATGAACATATTGCAAATTTAG
CTGGTAGCCCTGCTATTAAAAAAGGTATTTTACAGACTGTAAAAGTTGTTGATGAATTGGTCAA
AGTAATGGGGCGGCATAAGCCAGAAAATATCGTTATTGAAATGGCACGTGAAAATCAGACAACT
CAAAAGGGCCAGAAAAATTCGCGAGAGCGTATGAAACGAATCGAAGAAGGTATCAAAGAATTAG
GAAGTCAGATTCTTAAAGAGCATCCTGTTGAAAATACTCAATTGCAAAATGAAAAGCTCTATCT

CTATTATCTCCAAAATGGAAGAGACATGTATGTGGACCAAGAATTAGATATTAATCGTTTAAGT
GATTATGATGTCGATGCCATTGTTCCACAAAGTTTCCTTAAAGACGATTCAATAGACAATAAGG
TCTTAACGCGTTCTGATAAAAATCGTGGTAAATCGGATAACGTTCCAAGTGAAGAAGTAGTCAA
AAAGATGAAAAACTATTGGAGACAACTTCTAAACGCCAAGTTAATCACTCAACGTAAGTTTGAT
AATTTAACGAAAGCTGAACGTGGAGGTTTGAGTGAACTTGATAAAGCTGGTTTTATCAAACGCC
AATTGGTTGAAACTCGCCAAATCACTAAGCATGTGGCACAAATTTTGGATAGTCGCATGAATAC
TAAATACGATGAAAATGATAAACTTATTCGAGAGGTTAAAGTGATTACCTTAAAATCTAAATTA
GTTTCTGACTTCCGAAAAGATTTCCAATTCTATAAAGTACGTGAGATTAACAATTACCATCATGC
CCATGATGCGTATCTAAATGCCGTCGTTGGAACTGCTTTGATTAAGAAATATCCAAAACTTGAA
TCGGAGTTTGTCTATGGTGATTATAAAGTTTATGATGTTCGTAAAATGATTGCTAAGTCTGAGC
AAGAAATAGGCAAAGCAACCGCAAAATATTTCTTTTACTCTAATATCATGAACTTCTTCAAAAC
AGAAATTACACTTGCAAATGGAGAGATTCGCAAACGCCCTCTAATCGAAACTAATGGGGAAACT
GGAGAAATTGTCTGGGATAAAGGGCGAGATTTTGCCACAGTACGCAAAGTATTGTCCATGCCCC
AAGTCAATATTGTCAAGAAAACAGAAGTACAGACAGGCGGATTCTCCAAGGAGTCAATTTTACC
AAAAAGAAATTCGGACAAGCTTATTGCTCGTAAAAAAGACTGGGATCCAAAAAAATATGGTGGT
TTTGATAGTCCAACGGTAGCTTATTCAGTCCTAGTGGTTGCTAAGGTGGAAAAAGGGAAATCGA
AGAAGTTAAAATCCGTTAAAGAGTTACTAGGGATCACAATTATGGAAAGAAGTTCCTTTGAAAA
AAATCCGATTGACTTTTTAGAAGCTAAAGGATATAAGGAAGTTAAAAAAGACTTAATCATTAAA
CTACCTAAATATAGTCTTTTTGAGTTAGAAAACGGTCGTAAACGGATGCTGGCTAGTGCCGGAG
AATTACAAAAAGGAAATGAGCTGGCTCTGCCAAGCAAATATGTGAATTTTTTATATTTAGCTAG
TCATTATGAAAAGTTGAAGGGTAGTCCAGAAGATAACGAACAAAAACAATTGTTTGTGGAGCAG
CATAAGCATTATTTAGATGAGATTATTGAGCAAATCAGTGAATTTTCTAAGCGTGTTATTTTAG
CAGATGCCAATTTAGATAAAGTTCTTAGTGCATATAACAAACATAGAGACAAACCAATACGTGA
ACAAGCAGAAAATATTATTCATTTATTTACGTTGACGAATCTTGGAGCTCCCGCTGCTTTTAAAT
ATTTTGATACAACAATTGATCGTAAACGATATACGTCTACAAAAGAAGTTTTAGATGCCACTCT
TATCCATCAATCCATCACTGGTCTTTATGAAACACGCATTGATTTGAGTCAGCTAGGAGGTGACG
GCGGTGGAGGATCCGGAGGCGGTGGTAGCGGTGGAGGAGGCTCTTTGAAATATAATTTAACTGA
ATTTAAAAAAACAAAATCAAATATAAATAAAGCTTCACTGATTGGTTACAGTAAATTTAATTCA
GAAGGTTATGTTTATGATCCTGAATTTACAGGACCAACCTTAACTGCAAGCGGTGCAAATTCAA
GAATAAAAATCAAAGATGGATCTAATATTAGAAAAATGAACTCAGACGAAACTTTCTTATATAT
GGGGTTTGATTCACAAGATGGAAAAAGAGTAAATGAAATTGAATTTTTAACTGAAAATCAAAAA
ATATTTGTTTGTGGAAATTCAATATCAGTAGAAGTTTTGGAAGCGATTATAGATAAAATTGGAG
GTTAAGATACTTCTATTCTACTCTGACTGCAAACCAAAAAAACAAGCGCTTTCAAAACGCTTGTT
TTATCATTTTTAGGGAAATTAATCTCTTAATCCTTTTATCATTCTACATTTAGGCGCTGCCATCT
TGGGACAATGAAAACGTTAGTCATGGCGCGCCTTGACGGCTAGCTCAGTCCTAGGTACAGTGCTA
GCTTAATTAGTCTACGAGAACTTTCAGTTTAGCGGTCTGTTTTAGAGCTAGAAATAGCAAGTTA
AAATAAGGCTAGTCCGTTATCAACTTGAAAAAGTGGCACCGAGTCGGTGCTTTTTTTGAAGCTT
GGGCCCGAACAAAAACTCATCTCAGAAGAGGATCTGAATAGCGCCGTCGACCATCATCATCATCA
TCATTGAGTTTAAACGGTCTCCAGCTTGGCTGTTTTGGCGGATGAGAGAAGATTTTCAGCCTGAT
ACAGATTAAATCAGAACGCAGAAGCGGTCTGATAAAACAGAATTTGCCTGGCGGCAGTAGCGCG
GTGGTCCCACCTGACCCCATGCCGAACTCAGAAGTGAAACGCCGTAGCGCCGATGGTAGTGTGGG
GTCTCCCCATGCGAGAGTAGGGAACTGCCAGGCATCAAATAAAACGAAAGGCTCAGTCGAAAGA
CTGGGCCTTTCGTTTTATCTGTTGTTTGTCGGTGAACTGGA

<MN>

ATGAGCAAAGTAGAAAATAAAACAAAAAAACTTAGAGTATTTGAAGCTTTTGCTGGAATTGGTG
CTCAAAGAAAAGCCTTGGAGAAAGTCAGAAAAGATGAATATGAAATAGTAGGGCTTGCTGAATG
GTATGTTCCTGCAATTGTTATGTATCAAGCTATACACAACAATTTTCATACAAAGTTGGAGTAT
AAATCAGTTTCTAGAGAAGAAATGATTGACTATTTGGAAAATAAAACACTATCTTGGAACTCAA
AAAATCCAGTATCTAATGGTTATTGGAAGAGAAAAAAAGATGATGAACTTAAAATTATATATAA
TGCAATTAAGTTATCTGAAAAAGAGGGTAATATTTTTGATATTAGAGACCTTTACAAAAGAACT
TTGAAAAATATAGATTTATTAACATATTCATTTCCTTGTCAAGACTTATCTCAACAGGGTATTC
AAAAGGGTATGAAAAGAGGTTCTGGTACTAGATCAGGTCTCTTATGGGAAATTGAAAGAGCTTT
GGATTCAACTGAAAAAAATGACTTACCAAAATACTTGTTAATGGAAAATGTAGGGGCTCTTCTT
CACAAGAAGAATGAAGAAGAACTAAATCAATGGAAGCAAAAATTAGAAAGTCTTGGCTATCAAA
ACTCAATTGAAGTTTTGAATGCCGCTGACTTCGGTTCCTCACAAGCAAGAAGAAGAGTTTTTATG
ATATCTACTTTAAATGAATTTGTTGAACTACCAAAGGGAGATAAAAAACCTAAAAGTATCAAAA
AAGTTTTAAATAAAATAGTTTCTGAAAAAGATATTTTAAATAATTTATAA

Supplementary Text S2. DNA Sequences of dCas9-MC-hu and MN-hu for mammalian expression

KEY: dCas9 (purple) and MC (cyan) are joined by the 36 residue lfl linker (brown). An IRES sequence (grey) separates the MN-hu fragment (blue) from the dCas9-MC-hu. All NLS signals are shown in orange, the FLAG tag in red, and the HA tag in green.

<dCas9-MC-hu-IRES-MN-hu>
ATGCCAGTGAAGAGACCTGCTGCCACCAAGAAAGCTGGCCAGGCCAAAAAGAAGAAACTGGACT
ACAAAGACGATGACGACAAGACGCGTGATAAGAAATACAGCATAGGACTGGCCATTGGAACAAA
TTCTGTCGGCTGGGCGGTTATTACCGATGAATATAAGGTTCCGTCCAAGAAATTCAAGGTTCTAG
GCAATACAGACCGCCACAGTATTAAAAAGAATTTGATAGGTGCCCTTCTGTTTGACAGTGGCGA
GACAGCGGAAGCGACCAGACTCAAACGAACAGCCAGGAGACGTTATACAAGACGAAAGAATAGG
ATCTGTTATCTTCAAGAAATCTTTAGCAATGAGATGGCGAAAGTGGATGATAGTTTCTTTCATC
GCCTTGAAGAGTCCTTTCTGGTAGAAGAAGACAAGAAGCATGAAAGACATCCCATCTTTGGCAA
TATAGTGGATGAAGTTGCCTATCATGAGAAATATCCAACCATCTACCATCTACGCAAGAAACTG
GTGGATTCCACCGATAAAGCGGATCTGCGCCTGATCTATCTAGCACTAGCGCACATGATCAAGTT
TAGAGGGCATTTTCTGATCGAGGGCGATCTGAATCCCGATAATAGTGATGTAGACAAACTTTTC
ATTCAACTGGTGCAGACTTACAATCAGCTGTTTGAAGAAAACCCCATCAACGCTAGTGGCGTGGA
TGCCAAAGCGATCCTTTCCGCTCGCCTGAGTAAAAGCAGACGCCTGGAAAATCTCATCGCCCAAC
TCCCGGGGGAGAAGAAAAATGGACTGTTTGGTAACCTCATCGCCCTGAGCCTGGGGCTGACTCCC
AATTTTAAAAGCAATTTTGATCTGGCTGAAGATGCCAAACTGCAACTTAGCAAAGATACCTACG
ATGATGATCTGGATAATCTGCTGGCGCAGATCGGCGATCAGTATGCCGATCTGTTTCTGGCTGCC
AAGAATCTGAGCGATGCCATCCTGCTTAGCGATATTTTGAGAGTGAATACCGAAATAACCAAGG
CCCCGTTGAGCGCCAGCATGATCAAACGCTACGATGAACATCATCAGGACCTGACCCTTCTGAAA
GCCCTGGTTCGCCAGCAGCTTCCTGAAAAGTATAAAGAGATTTTCTTTGATCAGAGCAAGAACGG
CTATGCTGGGTATATCGATGGTGGCGCCTCTCAGGAAGAGTTCTATAAGTTCATTAAACCAATCC
TGGAAAAGATGGATGGGACCGAGGAACTGCTGGTAAAATTGAATAGGGAGGATCTGCTACGCAA
GCAGCGAACTTTTGACAACGGGTCCATCCCGCATCAGATCCACCTGGGGGAGCTACATGCCATCC
TGAGAAGACAGGAAGACTTTTATCCATTTCTGAAAGACAATCGGGAGAAGATCGAAAAGATTCT
GACCTTTCGCATCCCCTATTATGTTGGGCCTCTGGCGAGAGGAAACAGTAGGTTTGCTTGGATGA
CCCGAAAGTCCGAAGAAACAATCACTCCTTGGAACTTCGAAGAAGTTGTCGATAAAGGGGCCAG
CGCCCAGAGCTTTATCGAACGCATGACAAACTTTGATAAGAATCTTCCAAATGAAAAGGTGCTTC
CTAAACATAGTCTGCTTTATGAGTATTTTACGGTTTATAACGAACTGACAAAGGTCAAATATGT
TACCGAAGGAATGCGCAAACCAGCTTTTCTTAGCGGGGAACAAAAGAAAGCAATCGTTGATCTG
CTCTTCAAAACAAATCGCAAAGTGACTGTTAAGCAGCTGAAAGAAGATTATTTCAAGAAGATAG
AATGTTTTGATAGTGTTGAAATCAGCGGCGTTGAAGATAGATTCAATGCCAGCCTGGGGACTTA
CCATGATCTGTTGAAAATCATCAAGGATAAAGACTTCCTGGATAACGAAGAAAATGAGGATATT
CTGGAAGATATCGTTCTGACACTGACTCTGTTTGAAGATCGTGAGATGATCGAGGAAAGACTTA
AAACTTATGCCCACCTCTTTGATGATAAGGTAATGAAACAACTTAAAAGGCGCAGATATACCGG
GTGGGGCAGACTGTCCCGCAAACTGATCAATGGAATCCGTGATAAGCAGTCCGGAAAGACAATA
CTGGATTTTCTGAAAAGCGATGGGTTTGCAAATCGCAATTTTATGCAACTAATTCATGATGATA
GTCTGACATTCAAAGAAGACATCCAGAAAGCTCAGGTATCCGGCCAGGGAGATAGTCTGCATGA
ACATATCGCTAATCTGGCCGGGTCTCCCGCCATCAAGAAAGGAATCCTGCAAACCGTGAAAGTTG
TTGATGAACTGGTCAAAGTGATGGGTCGACATAAGCCAGAAAATATTGTTATCGAAATGGCTAG
AGAAAACCAAACAACCCAGAAGGGACAAAAGAACTCGCGCGAGAGGATGAAACGCATTGAAGAA

GGGATTAAAGAACTGGGCAGTCAAATCCTTAAAGAGCATCCCGTTGAAAATACCCAACTGCAAA
ACGAAAAGCTCTATCTCTATTATCTCCAGAATGGCAGAGACATGTATGTAGACCAGGAACTGGA
TATCAATAGGCTGAGTGATTATGATGTCGCCGCTATCGTTCCACAGAGTTTCCTTAAAGACGATA
GCATAGACAATAAGGTCCTGACGAGATCCGATAAGAATAGAGGGAAATCGGATAACGTTCCTAG
TGAAGAAGTGGTCAAGAAGATGAAGAACTATTGGAGACAGCTTTTGAACGCAAAGCTGATTACC
CAGAGGAAGTTCGATAATCTGACGAAAGCCGAAAGAGGCGGGCTGAGTGAACTTGATAAAGCCG
GGTTTATTAAACGCCAGCTGGTTGAAACCCGCCAGATTACCAAGCATGTAGCTCAGATCCTGGAT
AGTCGCATGAATACCAAATACGATGAAAATGATAAACTTATCCGCGAGGTTAAAGTAATCACTC
TGAAATCCAAACTGGTTTCCGACTTCCGCAAAGATTTCCAGTTCTATAAAGTGAGAGAGATCAAC
AATTACCATCATGCACATGATGCGTATTTGAATGCAGTCGTTGGCACCGCCCTGATCAAGAAGTA
TCCAAAACTTGAATCGGAGTTTGTCTATGGGGATTATAAAGTTTATGATGTTAGGAAAATGATC
GCCAAGTCCGAGCAGGAAATAGGAAAAGCTACTGCTAAGTATTTCTTTTACTCCAATATTATGA
ACTTCTTCAAAACAGAAATCACACTTGCTAATGGCGAGATCCGCAAACGCCCCTTGATTGAAACC
AATGGTGAAACCGGCGAAATCGTCTGGGATAAAGGTCGCGATTTTGCAACAGTACGCAAAGTGC
TGTCAATGCCGCAGGTCAATATCGTCAAGAAAACAGAAGTGCAAACAGGAGGCTTCTCAAAGGA
GAGCATCCTGCCTAAACGGAACTCGGACAAGCTTATCGCCAGGAAGAAAGACTGGGACCCAAAG
AAATATGGAGGGTTTGATAGTCCTACGGTGGCCTATAGCGTCCTTGTAGTTGCCAAGGTAGAGA
AGGGTAAGTCGAAGAAGCTGAAATCAGTTAAAGAGCTGCTTGGTATTACAATCATGGAAAGAAG
TTCATTTGAAAAGAATCCGATCGACTTTCTGGAAGCCAAAGGCTATAAGGAAGTTAAGAAAGAC
CTGATTATCAAACTTCCCAAATATAGTCTTTTCGAGCTGGAAAACGGGAGGAAACGAATGCTAG
CCAGTGCAGGCGAACTGCAGAAAGGCAATGAGCTAGCCCTACCATCTAAGTATGTAAACTTCCTG
TATCTGGCCAGTCATTATGAAAAGCTGAAGGGGAGTCCAGAAGATAACGAACAGAAGCAGCTGT
TTGTAGAGCAACATAAGCATTATCTGGATGAGATCATCGAGCAGATTAGTGAATTTTCCAAGAG
GGTTATCCTGGCTGATGCAAATCTGGATAAAGTTCTTAGTGCTTATAACAAACATAGAGACAAA
CCAATAAGGGAACAGGCTGAAAATATCATCCATCTGTTTACGCTGACGAATCTTGGCGCCCCAGC
CGCCTTCAAGTATTTCGATACAACAATCGATAGGAAACGCTATACGTCCACAAAAGAAGTTCTGG
ATGCAACCCTTATTCATCAGTCAATTACCGGGCTTTATGAAACACGCATCGATCTGAGTCAACTT
GGAGGGGACCCTGCTTCTGGCGGAACTTCATCTGATGGTGGCACGTCAGACGGAGGGTCAAGCAA
CACAGGCGGTAGCTCTGACGGAGGGAGCTCAGAAGGCGAACCTGCGCATGCACTGAAGTACAACC
TGACTGAGTTCAAGAAGACCAAATCAAACATCAACAAGGCAAGCCTCATTGGGTATAGCAAGTT
CAACTCAGAAGGCTACGTGTACGACCCTGAGTTCACAGGACCAACCCTGACTGCAAGTGGAGCAA
ACTCACGGATCAAGATCAAGGACGGATCTAACATTCGCAAGATGAACTCAGATGAAACGTTTCT
GTACATGGGCTTCGATTCACAAGATGGAAAGAGGGTGAACGAGATTGAGTTCCTGACTGAAAAC
CAGAAGATCTTCGTGTGCGGAAACTCAATCTCCGTGGAAGTTCTTGAAGCGATCATCGACAAGAT
TGGAGGGACCGGACCGAAGAAGAAACGGAAAGTGTAATCTAGATGATCAGAACGGTCAGCCTGC
TACGTAAATTCCGCCCCCCCCCCCTAACGTTACTGGCCGAAGCCGCTTGGAATAAGGCCGGTGT
GCGTTTGTCTATATGTTATTTTCCACCATATTGCCGTCTTTTGGCAATGTGAGGGCCCGGAAACC
TGGCCCTGTCTTCTTGACGAGCATTCCTAGGGGTCTTTCCCCTCTCGCCAAAGGAATGCAAGGTC
TGTTGAATGTCGTGAAGGAAGCAGTTCCTCTGGAAGCTTCTTGAAGACAAACAACGTCTGTAGC
GACCCTTTGCAGGCAGCGGAACCCCCCACCTGGCGACAGGTGCCTCTGCGGCCAAAAGCCACGTG
TATAAGATACACCTGCAAAGGCGGCACAACCCCAGTGCCACGTTGTGAGTTGGATAGTTGTGGA
AAGAGTCAAATGGCTCTCCTCAAGCGTATTCAACAAGGGGCTGAAGGATGCCCAGAAGGTACCCC
ATTGTATGGGATCTGATCTGGGGCCTCGGTGCACATGCTTTACATGTGTTTAGTCGAGGTTAAAA
AAACGTCTAGGCCCCCCGAACCACGGGGACGTGGTTTTCCTTTGAAAAACACGATGATAATATGG
CCACAACCATGCCTAAGAAGAAAAGAAAGGTTTACCCATACGACGTTCCAGACTACGCTAGTAAG
GTGGAGAACAAAACAAAGAAACTCCGGGTGTTCGAGGCTTTTGCTGGGATCGGGGCCCAACGGA

AGGCCTTGGAGAAAGTAAGGAAGGACGAGTATGAGATCGTTGGGCTTGCTGAGTGGTATGTCCC
TGCCATTGTGATGTATCAAGCGATCCACAATAACTTCCATACTAAGCTTGAGTACAAGTCCGTGT
CTAGGGAAGAGATGATTGATTACTTGGAGAACAAGACTCTATCTTGGAATAGCAAGAACCCCGT
GAGCAATGGCTACTGGAAGAGGAAGAAGGATGATGAACTAAAGATTATCTACAACGCTATAAAG
CTCTCAGAAAAGGAGGGGAATATCTTCGACATCAGGGATCTATATAAGCGGACTCTCAAAAACA
TTGACCTTCTGACATACAGCTTCCCTTGCCAGGATCTGTCCCAGCAAGGCATTCAGAAGGGTATG
AAAAGAGGATCTGGCACTAGGTCTGGACTCCTGTGGGAGATTGAAAGGGCACTGGACTCAACTG
AGAAGAACGATCTTCCAAAGTATCTATTGATGGAGAACGTTGGCGCTTTACTCCATAAGAAGAA
CGAGGAAGAGCTAAACCAGTGGAAACAGAAACTTGAGTCTTTGGGATACCAAAACTCAATTGAG
GTGTTGAACGCTGCCGATTTTGGCTCCTCTCAGGCTAGAAGGAGAGTGTTCATGATAAGCACTCT
TAACGAGTTCGTTGAACTGCCTAAAGGCGACAAGAAGCCTAAGTCCATCAAGAAAGTTCTGAAC
AAGATCGTGTCAGAGAAGGACATTCTGAACAACCTGTAA

Supplementary Text S3. HEK293T promoter sequences for SALL2 P2 and HBG1

SALL2 P2
Key: ATG translation start site (Red), CpG sites shown in bold.

TTTATCCAAAGAAAAATAAGAGAAGCCTAT**CG**CCTTGCTCCACTGAGCAGGGCTA**CG**TTTCCCAC
TGTGCCCCTAGAGCTTAGGAGGGGAGGG**CG**GGGGGAGGAGGGTAGGCCTGGGCAAAGGCATCTG
CAGATGGGGGGAGCTGGCCAGGGAC**CG**GGAAAGGTGGACCCC**CG**CAGTGCTTCACAGCACCTGTC
ACTG**CG**CCTC**CG**GAAACTCTCC**CG**AGCTCTGAGCTCC**CG**CCCCCA**CG**CCCC**CG**CTAG**CG**CTCCCTT
CTCC**CG**CCTCCTCTAGCTCC**CG**CCTCCCTCCCCAGCAGCTCCTCCCCATAAACTCCCCTCCTCATCA
GCTCCTCCCAGATCTCCCCTCTCCC**CG**CTTTCTCACTCCAGCTTCTCCC**CG**CCTCCT**CG**CTCCCCTC
CCCCTC**CG**C**CG**CTGCTCCCTGCCCC**CG**C**CG**CTGC**CG**ATCTCCATCTC**CG**CAGTCTGGGC**CG**CTGGG
TGCAGAGGCTGC**CG**CAGACCCAG**CG**GCCAT**CG**CCTCCCTCAATCCCAATATCCATTGTCTCCCACC
CCACCTGGCCCTCCACTTCCCCCACAACCATG

HBG1 promoter
Key: ATG translation start site (Red) and HBG1 exon 1 shown as underlined sequence. CpG sites are shown in bold. SNPs and mutations are shown as either Grey shaded sites indicating known HBG1/HBG2 SNP sites or a red shaded site showing mutation in the HEK293T cell line.

GGAATGACTGAAT**CG**GAACAAGGCAAAGGCTATAAAAAAAATTAAGCAGCAGTATCCTCTTGGG
GGCCCCTTCCCCACACTATCTCAATGCAAATATCTGTCTGAAA**CG**GTCCCTGGCTAAACTCCACC
CATGGGTTGGCCAGCCTTGCCTTGACCAATAGCCTTGACAAGGCAAACTTGACCAATAGTCTTAG
AGTATCCAGTGAGGCCAGGGGC**CG**G**CG**GCTGGCTAGGGATGAAGAATAAAAGGAAGCACCCTTC
AGCAGTTCCACACACT**CG**CTTCTGGAA**CG**TCTGAGGTTATCAATAAGCTCCTAGTCCAGA**CG**CC**A**
TGGGTCATTTCACAGAGGAGGACAAGGCTACTATCACAAGCCTGTGGGGCAAGG

Supplementary Text S4. U6-sgRNA cassette DNA sequence (for SALL2 R1 sgRNA). Key: U6 promoter is shown in purple, the R1 target sequence is shown in red and remaining sgRNA is shown in blue.

<U6-R1 sgRNA>
GAGAGGGAGTGGCCAACTCCATCACTAGGGGTTCCGATCCGACGCCGCCATCTCTAGGCCCGCGC
CGGCCCCCTCGCACAGACTTGTGGGAGAAGCTCGGCTACTCCCCTGCCCCGGTTAATTTGCATAT
AATATTTCCTAGTAACTATAGAGGCTTAATGTGCGATAAAAGACAGATAATCTGTTCTTTTTAA
TACTAGCTACATTTTACATGATAGGCTTGGATTTCTATAAGAGATACAAATACTAAATTATTAT
TTTAAAAAACAGCACAAAAGGAAACTCACCCTAACTGTAAAGTAATTGTGTGTTTTGAGACTAT
AAATATCCCTTGGAGAAAAGCCTTGTTTGACAATGGATATTGGGATTGGTTTTAGAGCTAGAAA
TAGCAAGTTAAAATAAGGCTAGTCCGTTATCAACTTGAAAAAGTGGCACCGAGTCGGTGCTTTT
TTTCTCGAGGTCGACGGTATCGATAAGCTCGCTTCACGAGATTCCAGCAGGTCGAGGGAC
